# Emerging Issues on Tropane Alkaloid Contamination of Food in Europe

**DOI:** 10.3390/toxins15020098

**Published:** 2023-01-19

**Authors:** Monique de Nijs, Colin Crews, Folke Dorgelo, Susan MacDonald, Patrick P. J. Mulder

**Affiliations:** 1 Wageningen Food Safety Research, Wageningen University & Research, 6708 WB Wageningen, The Netherlands; 2 Fera Science Ltd., York YO41 1LZ, UK

**Keywords:** tropane alkaloids, food, calystegines

## Abstract

The occurrence of tropane alkaloids (TAs), toxic plant metabolites, in food in Europe was studied to identify those TAs in food most relevant for human health. Information was extracted from the literature and the 2016 study from the European Food Safety Authority. Calystegines were identified as being inherent TAs in foods common in Europe, such as *Solanum tuberosum* (potato), *S. melongena* (eggplant, aubergine), *Capsicum annuum* (bell pepper) and *Brassica oleracea* (broccoli, Brussels sprouts). In addition, some low-molecular-weight tropanes and Convolvulaceae-type TAs were found inherent to bell pepper. On the other hand, atropine, scopolamine, convolvine, pseudotropine and tropine were identified as emerging TAs resulting from the presence of associated weeds in food. The most relevant food products in this respect are unprocessed and processed cereal-based foods for infants, young children or adults, dry (herbal) teas and canned or frozen vegetables. Overall, the occurrence data on both inherent as well as on associated TAs in foods are still scarce, highlighting the need for monitoring data. It also indicates the urge for food safety authorities to work with farmers, plant breeders and food business operators to prevent the spreading of invasive weeds and to increase awareness.

## 1. Introduction

Tropane alkaloids (TAs) are secondary metabolites inherent to several plant families, including Brassicaceae, Convolvulaceae, Erythroxylaceae and Solanaceae [1]. TA-containing food plants are found in these families, such as *Solanum tuberosum* (potato), *Capsicum annuum* (bell pepper), *Solanum melongena* (eggplant or aubergine) and *Ipomoea batatas* (sweet potato) [2,3]. Some TA-containing weed plants that grow in food crop systems, such as *Datura* and *Atropa belladonna*, also belong to these families. TAs from weeds contaminate food crops when the weed is co-harvested with the food crop. The inherent TAs occurring in food plants are mainly calystegines. The types of TAs occurring in the non-food but co-harvested weed plants are typically esters of hydroxytropanes with short-chain acids; however, the presence of calystegines in these weed plants cannot be excluded [4].

TA-producing plants in general generate a profile of TAs correlating to the genetic make-up of the plant family [5,6,7,8,9]. Growth state, plant part, cultivar and environmental factors such as climate or handling of the product influence the final concentration of the TA [10,11]. In general, it is believed that the production of TAs is a defense mechanism of the plant to prevent disease, grazing or insect damage [12].

The biosynthesis of TAs proceeds from ornithine (see Figure S1) that is converted in several steps to tropinone, the central compound from which all TAs relevant in this review are derived [4,7,13,14]. Tropinone is converted by enzymes into tropine (3α-tropinol) by tropinone reductase TR I or into pseudotropine (3β-tropinol) by tropinone reductase TR II. The most well-known TAs, (-)-hyoscyamine, atropine (a racemic mixture of (−)-hyoscyamine and (+)-hyoscyamine), and (-)-scopolamine, are formed by the esterification of tropine with phenyllactic acid to produce littorine, which in turn isomerizes to (-)-hyoscyamine. Subsequently, (-)-hyoscyamine can be transformed in two steps, catalyzed by hyoscyamine 6β-hydroxylase (H6H) to (-)-scopolamine (see Figure S1) [15,16]. A wide range of 3α-esters can be produced from tropine by the action of tropine acyltransferases (TAT) and 3β-esters from pseudotropine by pseudotropine acyltransferases (PAT). Calystegines are polyhydroxy alkaloids with a nortropane skeleton that originates from pseudotropine; however, the metabolic pathway has yet to be described [2,4,7,14].

Based on their chemical structure, the major TA group is identified by atropine, scopolamine and related compounds (see Figure S2). The group of Convolvulaceae-type TAs, such as convolamine and convolidine, are tropane esters derived from benzoic acid (see Figure S3), while the group of low-molecular-weight tropane esters is derived from acetic acid or tiglic acid (see Figure S4). The fourth group is composed of some low-molecular-weight (LMW) tropanes (see Figure S5). The polyhydroxylated calystegines form a separate group of TAs (see Figure S6).

The impact on human health of the TAs occurring in food in Europe is largely unknown due to a lack of occurrence data [1,17,18,19,20,21,22,23,24]. Even though over 200 TAs are known, only atropine and (-)-scopolamine are mentioned when intoxications related to food are described [4,25]. Atropine and scopolamine are anticholinergic agents, blocking the binding of the neurotransmitter acetylcholine to the muscarinic acetylcholine receptors [26]. They have an effect on the peripheral nervous system (PNS) in humans, expressed as dryness of the mucous membranes in the upper respiratory and digestive tracts (i.e., dry mouth), pupil dilation, urinary and gastric retention, and effects on heart rate [4]. The central nervous system (CNS) is also affected, leading to ataxia, sleepiness, speech disturbance, lack of concentration, hallucinations and a decrease in heart rate and blood pressure [4]. On account of their adverse health effects and their regular occurrence in food in Europe, atropine and scopolamine are regarded as undesirable substances in food and feed and for that reason have been subjected to European Union (EU) regulation since 2016 [27,28,29]. No known incidents in humans related to calystegines present in food have been reported [30]. However, it is known that calystegines can inhibit glucosidase activity, the enzymes that break down oligosaccharides into glucose molecules. As mentioned in the EFSA Report of 2019 on calystegines, experimental models for these kind of toxic effects are not available [30].

The review presented here aims to describe the current knowledge on the occurrence of TAs in foods in Europe and to identify emerging issues. Reports on TAs for recreational use were excluded from the study. The description of the adverse effects of TAs to the health of humans and animals was excluded from this study, except for some brief information in the introduction section, since no literature on other TAs than atropine and scopolamine related to food was retrieved. This review is partly based on the literature study and survey on TAs in food in Europe carried out for the European Food Safety Authority (EFSA) and published in 2016 [31].

## 2. TAs in Food in Europe

This section presents the occurrence of TAs in foods consumed in Europe containing inherent TAs (Section 2.1) and for TA-containing weeds that are co-harvested and thus contaminating food in Europe (Section 2.2). Notifications from the Rapid Alert System for Food and Feed (RASFF) are described in Section 2.3, while in Section 2.4 a short overview of human intoxications related to foods in Europe contaminated with TAs is given. Factors influencing the exposure of humans to TAs through food are described in Section 2.5.

### 2.1. TA-Containing Food Plants

Foods containing inherent TAs and consumed in the EU mainly belong to the plant families of Brassicaceae (Section 2.1.1), Convolvulaceae (Section 2.1.2), Moraceae (Section 2.1.3) and Solanaceae (Section 2.1.4). The food plants of these families as mentioned in the literature are described below in alphabetical order, and, if available, information on the TA concentration is provided. The information is summarized in Table 1 together with information on some other less-important TA-containing food plants. Results from surveys on the occurrence of inherent TAs in food plants (Section 2.1.5) as well as the stability of the inherent TAs during processing (Section 2.1.6) are described.

#### 2.1.1. Brassicaceae Food Plants

Many Brassicaceae plants are staple foods of the European diet, particularly those from the species *B. oleracea* such as broccoli, cauliflower, kale, kohlrabi and Brussels sprouts. Other commonly consumed Brassicaceae plants are *B. rapa* (turnip, white turnip) and *B. napobrassica* (Chinese cabbage, swede, rutabaga). Some Brassicaceae plants are consumed to a lesser but growing extent in Europe, such as *Eruca sativa* (rocket, arugula), *Raphanus sativus* (radish) and *Nasturtium officinale* (watercress). Some Brassicaceae are consumed as ingredients or as spices, such as *B. juncea* (brown mustard), *B. nigra* (black mustard) and *Sinapis alba* (white mustard), or as sauce components such as *Armoracia rusticana* (horseradish) and *Wasabi japonica* (wasabi).

Calystegines are accumulated throughout the life cycle of Brassicaceae plants and are found in all parts of the plant [32]. Some Brassicaceae (*B. nigra* and *B. oleracea*) contain calystegines A_3_, A_5_ and B_2_, with a maximum of 25 mg calystegine A_3_/kg dry weight (d.w.) in the leaves of *B. oleracea* [32].

Food plants from the *Brassica* genus, consumed in small quantities in the European diet, can also contain TAs [32]. Calystegines A_3_, A_5_ and B_3_ were detected in *Crambe maritima* (sea kale) and calystegines A_3_ and A_5_ in *Lepidium sativum* (garden cress) [32].

#### 2.1.2. Convolvulaceae Food Plants

The most relevant food plants of the Convolvulaceae family belong to the genus *Ipomoea*, of which about 700 species can be found in most tropical and subtropical regions. A well-known food plant of this family is the sweet potato *Ipomoea batatas.* Sweet potatoes are grown in many countries, and the tubers are consumed throughout most of the world [37,38]. *Ipomoea aquatica* (water spinach) is a food crop in much of Asia, where the leaves are considered a rich source of nutrients and essential amino acids [39].

A survey on the occurrence of TAs in 129 Convolvulaceae species (food crops and weeds) revealed the presence of one to six calystegines in sixty-two species belonging to twenty-two genera [7]. Calystegines A_3_, B_1_, B_2_ and C_1_ have been detected in *I. batatas* [5]. In the tuber of *I. batatas* the following calystegines levels were reported: A_3_, 0.11; B_1_, 2.4–16; B_2_, 1.1–19; and C_1_, 0.61–9 mg/kg fresh weight (f.w.) [5,36]. Calystegines A_3_, B_1_, B_2_ and B_4_ were reported in *I. aquatica* from Thailand [7].

#### 2.1.3. Moraceae Food Plants

The fruit of *Morus alba* (white mulberry) is consumed in Asia and the USA and is becoming more popular in Europe, although the *M. rubra* (red) and *M. nigra* (black) species are preferred [40]. *Morus alba* is a tree of economic importance in Asia for the silk industry; the young leaves are sometimes consumed by humans as vegetables. *M. alba* is considered a weed in the USA. In the fruits of *M. alba,* a total of 10 polyhydroxylated alkaloids were detected [33]; of these, calystegine B_2_ was present at a level of 7 mg/kg d.w. [5,36].

#### 2.1.4. Solanaceae Food Plants

Most of the food plants with inherent TAs belong to the Solanaceae family. It is one of the most diverse and varied groups of plants, with 90 genera and just over 4000 species. It is found worldwide, with the majority of species in Central and South America. Food plants of the Solanaceae family consumed in Europe belong to *Capsicum*, *Lycium* and *Solanum*. *C. annuum* (bell pepper) is included in many European diets. *Solanum tuberosum* (potato) originated from South America and has become a staple food in the European diet since the 18th century. Also, *S. melongena* (eggplant) has become part of the European diet, and it has been cultivated in the Mediterranean area since the 7th century. Several related species are grown on a small scale for food, including scarlet and gboma eggplants (*S. aethiopicum* and *S. macrocarpon*). The consumption of *Physalis philadelphica* (syn. *P. ixocarpa*) (tomatillo) has increased in popularity in Europe as a result of an interest in Mexican food. *P. peruviana* (Cape gooseberry) is consumed in large areas of Europe, although in small quantities. The roots and leaves of *Lycium* have a history of use for medicinal purposes in China, but in the Western world only the berries of *L. barbarum* (goji berry) are marketed as foods beneficial to health [41].

Of the domesticated *Capsicum* species, *C. annuum* (bell pepper) and four chili-types, *C. baccatum*, *C. chinense*, *C. frutescens* and *C. pubescens*, are important food plants. The fruits of *C*. *annuum* have been found to contain calystegines B_1_ (12 mg/kg f.w.), B_2_ (37 mg/kg f.w.) and C_1_ (4 mg/kg f.w.), but no A_3_ was detected [5,36]. *C. frutescens* grown in the UK contained calystegine A_3_ (0.24 mg/kg f.w.) and B_2_ (0.27 mg/kg f.w.) but no B_1_ or C_1_ [5,36].

The fruits from *Lycium barbarum* (Indian goji berry) in 1989 were reported to contain atropine at a level of 9500 mg/kg dry weight and hyoscyamine at 2900 mg/kg dry fruit [42]. Later, it was concluded by Peng (2005) that the original test was not specific for tropanes and that the plant was probably misidentified as *Lycium europaeum* [34,43]. Peng (2005) analyzed the berries of both *L. barbarum* and *L. europaeum* from China by Liquid Chromatography-Tandem Mass Spectrometry (LC-MS/MS) [43]. Atropine was measured at a concentration of 0.59 mg/kg d.w. in the fruits of *L. europaeum,* and it was present at the detection limit (0.01 mg/kg) in the fruits of *L. barbarum* [43]. A study on goji berries from China and Thailand detected atropine at trace levels only in one of eight samples of dried berries (0.019 mg/kg d.w.), whereas for the seven other samples, the level was <0.01 mg/kg d.w. [44]. Zhao and Shi (2020) investigated 30 goji berry batches from different production areas in China and reported the presence of trace levels of atropine in 29 of the samples (below the Limit of Detection (<LOD-23.4 µg/kg d.w.). Scopolamine and anisodamine could not be detected in the samples (LOD 1.5 µg/kg d.w.) [45].

The roots and/or leaves of *Physalis peruviana* (Cape gooseberry) and the related *P. alkekengi* (Chinese lantern, bladder berry) contain several tropane and secotropane alkaloids [6]. The roots of *P. alkekengi* contain a total of 840–1040 mg/kg TA. The roots of *P. peruviana* also contain the secotropane alkaloids physoperuvine and (+)-N,N-dimethyl-physoperuvinium salt [46]. The leaves and roots contain tropine, 3α-tigloyloxytropane, 3β-acetoxytropane and physoperuvine [47]. The roots of the related *P. alkekengi* contain the calystegines A_3_, A_5_, B_1_, B_2_ and B_3_ [48]. An analysis of the fruits of *P. peruviana* revealed the presence of low levels of calystegines A_3_ (0.003 mg/kg f.w.), B_1_ (0.038 mg/kg f.w.), B_2_ (0.048 mg/kg f.w.) and C_1_ (0.005 mg/kg f.w.) [5,36]. The cultured roots of *P. philadelphica*, the tomatillo, have been shown to produce calystegine B_2_ but not A_3_, A_5_, or B_1_, which were however present in cultured roots of the related species *P. divaricata* and *P. pubescens* [49].

Low levels of calystegine B_2_ (0.002 mg/kg f.w.) were detected in the fruit of *S. betaceum* (syn. *Cyphomandra betaceae*) (tamarillo, tree tomato) [5,36], and trace levels were found in *S. melanocerastrum* (syn. *Solanum scabrum*) (garden huckleberries) [5,36].

Calystegines were reported in the fruits of *Solanum esculentum* (syn. *Lycopersicum esculentum)* (tomato plant), calystegine A_3_ at 1.1 mg/kg and calystegine B_2_ at 4.5 mg/kg [5,36]. Calystegine A_3_ (<0.5–5.43 mg/kg f.w.), A_5_ (<0.5–5.01 mg/kg f.w.), B_2_ (<0.5–12.47 mg/kg f.w.) and C_1_ (<0.5–2.39 mg/kg f.w.) were detected in nine tomato varieties grown in a greenhouse in the southeast of Spain [9].

Fruits of *S. melongena* (eggplant) grown in the UK contained calystegine A_3_ at 0.3 and B_2_ at 0.5 mg/kg f.w., while fruits obtained from Japan contained calystegine B_1_ at 7 and B_2_ at 73 mg/kg f.w. [5,36].

Various calystegines were detected in more than 70 edible varieties of *S. tuberosum* (potato) [11,35,36,50]. Calystegine A_3_ and B_2_ were reported in potatoes obtained in the UK and calystegine B_2_ in potatoes obtained from Japan in the range of 1.17 to 7 mg/kg total tuber f.w. [5,36]. The ratio of calystegine B_2_:A_3_ in the potatoes under investigation was typically 2:1 [36]. Calystegines A_3_ and B_2_ were detected in seven potato-based products from UK supermarkets (crisps, fries, mashed potatoes and oven products) in the range of 0.21 to 15.16 mg/kg for calystegine A_3_ and 0.3 to 22.66 mg/kg for calystegine B_2_ [36,50]. In a broader study on the effect on tuber stress conditions (light, heat and mechanical wounding), Petersson et al. (2013) studied the calystegine levels in 13 potato cultivars [11]. The reported average (range) levels in the control group were calystegine A_3_: 33 (9–84) mg/kg f.w.; B_2_: 32 (10–56) mg/kg f.w.; and B_4_: 7 (<1–30) mg/kg f.w. The average sum of the three calystegines was 72 mg/kg and ranged between 25 and 141 mg/kg f.w.

#### 2.1.5. Surveys on Inherent TAs in Food

Surveys on TAs inherent to food plants available at retail stores in Europe are scarce. Two references on surveys in food in Europe were identified—one study with a limited number of samples published in 1997 and the EFSA study on the occurrence of TAs in food in the EU published in 2016.

A study was identified in which seven samples of commercially available potato products were analyzed for calystegines [36]. Calystegine A_3_ was detected in the range of 0.21 to 4.89 mg/kg and calystegine B_2_ between 0.30 and 20.97 mg/kg in crisps made from freeze-dried potato granules or whole potato. Microwave fries and instant mash potato granules contained calystegine A_3_ and B_2_ in concentrations of 15.16 and 19.53 mg/kg and 8.96 and 22.66 mg/kg, respectively.

Details on the EFSA study [31] are presented in Table S1 and discussed in this paragraph. A total of 404 samples of potato (including processed potato foods), eggplant and bell pepper available at retail stores in Europe were collected in 2016 and analyzed for calystegines A_3_, A_5_, B_1_, B_2_, B_3_ and B_4_ [31]. One or more calystegines were detected above the LOD (range 0.25 to 1 mg/kg f.w.) in all potato samples, in 96.7% of the eggplant samples and in 33% of the bell pepper samples. The mean concentration of calystegines in the 297 potato samples was 164.0 mg/kg f.w. with a maximum of 507.3 mg/kg f.w. The predominant calystegine found was A_3_ (average 108.3 mg/kg f.w.), followed by B_2_ (average 52.1 mg/kg f.w.). Calystegine B_4_ was present in much lower concentrations (average 3.7 mg/kg f.w.) and occasionally traces of A_5_ and B_3_ were found. The calystegine concentrations in 11 processed potatoes samples were substantially lower, with a mean of 95.6 mg/kg f.w. and a maximum concentration of 207.7 mg/kg f.w. In eggplant, calystegines A_3_, B_1_ and B_2_ were detected most frequently, with calystegine B_2_ at the highest mean concentration (14.5 mg/kg f.w.), followed by B_1_ (3.9 mg/kg f.w.) and A_3_ (2.7 mg/kg f.w.) The other calystegines (A_5_, B_1_, B_4_) were practically absent. The mean calystegine concentration in the 90 eggplant samples was 21.1 mg/kg f.w. with a maximum of 181.5 mg/kg f.w. The six samples of bell peppers contained only traces of calystegine B_1_ at a mean concentration of 0.2 mg/kg f.w. and a maximum of 0.5 mg/kg f.w.

In the product categories ‘legumes and stir-fry mixes’ and ‘pasta and cereal-based meals for children’, several LMW tropanes were detected. Tropine was always present in the highest concentration (up to 2000 µg/kg in a vegetable stir-fry mix), but pseudotropine (up to 174 µg/kg) and smaller amounts of tropinone, nortropinone and 6-hydroxytropinone were also found in these samples. Convolvulaceae-type TAs, including convolidine, convolvine, phyllalbine and convolamine, were also detected in the same products, albeit at lower concentrations (up to 40 µg/kg for the sum of the 4 TA). The presence of these TAs seems correlated to the use of bell pepper as an ingredient in vegetable products. These results indicate that bell pepper can produce a wide set of TAs, including calystegines, LMW tropanes as well as Convolvulaceae-type TAs.

#### 2.1.6. Stability of Inherent TAs during Storage and Processing

After 5 months of storage of 21 varieties of potatoes, the total calystegine level in the tubers was not affected by wounding or light exposure [11]. Calystegine levels in tuber peel, however, showed an increase when the tubers were stored after harvest for 5 months, while the concentration slightly decreased after 8 months [51].

Watson et al. (2000) reported that all seven processed potato products collected from retail (potato waffles, oven ready chips, instant mashed potato granules, crisps made from whole potato, crisps made from dried potato granules and hula hoops) contained calystegines A_3_ and B_2_, which indicates a certain resistance of calystegines to processing [36]. The same authors found that processing potatoes by boiling, oven roasting, microwave preparation or deep frying decreased the calystegine content to 15–20% of the original concentration [36].

The stability of TAs to typical processing conditions was also shown in the EFSA study [31]. TAs were detected in processed cereal-based foods such as bread, biscuits for children and ready-to-eat pasta and cereal-based meals for children.

### 2.2. TAs from Associated Weed Plants in Food of Plant Origin

Food is contaminated with TAs when weed plants that contain inherent TAs are co-harvested. The occurrence of TAs in the weed plants of the families Brassicaceae (Section 2.2.1), Convolvulaceae (Section 2.2.2) and Solanaceae (Section 2.2.3) are described in alphabetical order below. The information is summarized in Table 2, together with information on some other less important associated weeds. Surveys on TAs from associated weeds in foods available on the European market are described (Section 2.2.4) as are the influence of processing on TA stability (Section 2.2.5). Finally, an overview is presented on the emerging issues related to weeds growing in the food crops during the field period (Section 2.2.6).

#### 2.2.1. Brassicaceae Weed Plants

Leaves from *Brassica campestris* (wild turnip), a pernicious weed, were reported to contain calystegines A_3_ at 17 mg/kg d.w. and A_5_ at 3 mg/kg d.w. [32].

*Camelina sativa* (false flax), a specific weed of flax, contains calystegines A_3_, A_5_, B_2_ and B_3_ at levels of 1 to 5 mg/kg [32]. *C. sativa* is increasingly used for the production of biodiesel in USA and Europe [74].

*Cochlearia* species (scurvy grass) are annual Brassicaceae plants that do not have an impact on crops but were the first Brassicas to be shown to contain the TAs tropine and pseudotropine (concentrations up to 60 mg/kg d.w. in leaves) as well as calystegines A_3_, A_5_, B_3_ in levels up to 5000 mg/kg d.w. in leaves [32].

#### 2.2.2. Convolvulaceae Weed Plants

*Convolvulus arvensis* (field bindweed) is considered a highly invasive weed [75]. Early studies reported tropinone and feruloyltropanol to occur in five Convolvulaceae species, among which was *C. arvensis* [76]. The fresh aerial parts of *C. arvensis* plants, related to poisoned horses in Colorado USA, were shown to contain pseudotropine and traces of tropine and tropinone but no calystegines [52]. However, Schimming et al. (2005) reported calystegines A_3_, A_5_, B_2_, B_3_ and B_4_ in the aerial parts and calystegines A_3_, A_5_, B_1_, B_2_, B_3_ and B_4_ in the flowers of Convolvulaceae [7]. The roots of *C. arvensis* were reported to contain calystegines A_3_, B_1_, and B_2_ [77].

Other TAs, such as convolvine (3α-veratroyloxynortropane), convolamine (3α-veratroyloxytropane), convolidine (3α-vanilloyloxynortropane), confoline (3α-veratroyl-*N*-formylnortropine), convolamine-*N*-oxide and confolidine ((±)3α-vanillyl-*N*-formylnortropane) have been detected in various other *Convolvulus* spp. such as *C. lineatus* and *C. subhirsutus* [77].

#### 2.2.3. Solanaceae Weed Plants

TAs are present in all parts of the weed plant *Atropa belladonna* (deadly nightshade), and total TA levels can vary considerably between different cultivars and harvesting stages. Nevertheless, they comprise mainly atropine and some scopolamine both in leaves and seeds [10,56]. *A. belladonna* plants from Iran contained about 550 mg/kg d.w. TAs (sum of atropine and scopolamine) in the leaf, 588 mg/kg d.w. in the root and 948 mg/kg d.w. in the stem. Seeds of cultivated *A. belladonna* contained 692 mg TAs (sum of atropine and scopolamine)/kg d.w. [10]. The dried leaves of *A. belladonna* contained atropine ranging from 1100 to 2400 mg/kg and scopolamine from 3 to 10 mg/kg, while the berries contained atropine ranging from 160 to 740 mg/kg but no scopolamine [57]. An overview of the quantitative data available in the literature on the occurrence of TAs in *A. belladonna* is given in the EFSA opinion on TAs [4]. The sum of atropine and scopolamine were reported in the roots of *A. belladonna* ranged from 500–4000 mg/kg d.w., in the leaf from 700–5100 mg/kg d.w. and between 1300–7300 mg/kg d.w. in the seed [4]. Several other minor TAs have been reported in *A. belladonna* plants such as tropine, belladonnine, norhyoscyamine, apoatropine and 6β-hydroxyhyoscyamine in leaf extracts [53]; tigloyltropeine, aposcopolamine, apoatropine, hydroxyhyoscyamine and tigloyloxytropane in leaves and berries [56]; and littorine in roots [58]. Several authors reported the presence of calystegines in *A. belladonna* [55]. Calystegine A_3_ was located in all plant parts but mainly in the young upper leaves in a concentration of 40–60 mg/kg f.w. [14]. Calystegine A_3_, B_1_, B_2_ and B_3_ were reported in the aerial parts of *A. belladonna*, with levels of up to 100 mg calystegine B_2_/kg d.w. [54].

The *Datura* species are a frequent contaminant of food crops as is evidenced by the reports under the EU RASFF system (described in Section 2.3). The TA profiles of the *Datura* species have been studied in detail, and more than 65 different TAs have been reported [62,63]. Most of the information, however, relates to atropine and scopolamine, which predominate in all *Datura* species investigated [8,63,64]. TA patterns in *Datura* are influenced more strongly by environmental factors than by genetic ones, but the proportion of the less-common TAs in the seed of European *Datura* is small compared to those of atropine and scopolamine [63]. A total of 14 TAs were reported in the leaves of three *Datura stramonium* varieties, of which apoatropine, hyoscyamine, aposcopolamine and scopolamine occurred in all three cultivars [63]. The EFSA summarized the available data on TA sum concentrations in *D. stramonium*, with maximum levels of 3400 mg/kg d.w. in the seeds, 6430 mg/kg d.w. in the leaves, 6710 mg/kg d.w. in the flowers and 8830 mg/kg d.w. in the stem [4]. Tsialtas et al. (2018) reported atropine at up to 39.27 mg/kg d.w. and scopolamine at up to 14.85 mg/kg d.w. in the leaves and atropine at 2.87 mg/kg d.w. and scopolamine at 0.51 mg/kg d.w. in the fruits [66]. Higher levels of TAs were found by Sramska et al. (2017) in *D. stramonium*: the leaves contained atropine at 123.0 mg/kg d.w. and the stems at 1282.6 mg/kg d.w. [65]. Scopolamine was present at 1081.3 mg/kg d.w. in the leaves and at 1014.1 mg/kg d.w. in the stems [65]. The dried leaves of *D. stramonium* contained 161.7 mg scopolamine/kg and 222.1 mg atropine/kg in the juvenile stage, while in the reproductive stage during flowering, the levels of scopolamine increased to 634 mg/kg and that of atropine to 694.3 mg/kg [12]. The same authors showed that the levels of TAs vary largely between the *Datura* species in the reproductive stage, e.g., the dried leaves of *D. ferox* in the reproductive stage contained 1015 mg scopolamine/kg d.w. and the dried leaves of *D. metel* had 2503 mg scopolamine/kg d.w.

*Duboisia leichhardtii* is a tree cultured in Australia, of which the leaves contain calystegines B_1_, B_2_, B_4_, C_1_ and C_2_ [2,69]. *D. leichhardtii* and *Duboisia myrporoïdes* contain high concentrations of atropine and scopolamine, but the trees are too few in number to cause a weed problem [13,56].

Atropine and scopolamine have been detected in *Hyoscyamus albus* (white henbane) [56] as well as calystegines A_3_, B_1_, B_2_ and B_3_, with calystegine B_2_ present at the highest concentration (75 mg/kg d.w.) in the leaves [54].

*Hyoscyamus niger* (black henbane) is a weed of poppy, wheat and millet crops with atropine and scopolamine as the major TA [56,78]. *H. niger* from Iran can contain levels of both TAs as high as 700 mg/kg d.w. in the stem, 1045 mg/kg d.w. in the leaf, 2240 mg/kg d.w. in the flower and 2980 mg/kg d.w. in the seed [4,70]. In addition, plants of this species may also contain calystegines: A_3_, A_5_, A_6_, B_1_, B_2_, B_3_ and N_1_ [2].

*Mandragora autumnalis* (autumn mandrake) is a Mediterranean species that can contain many TAs [71], but it is not particularly effective as a weed. However, a number of human poisonings by *M. autumnalis* have been reported in Italy [79,80,81]. The roots of *M. autumnalis* and its close relative *Mandragora vernalis* syn. *M. officinarum* (mandrake) contain hyoscyamine, hyoscine, apoatropine, 3 α-tigloyloxytropane and 3,6-ditigloyloxytropane, while belladonnine was present in the dried roots but could not be detected in fresh roots [72]. The leaves of *M*. *autumnalis* contain about 20 mg calystegine B_2_/kg d.w. and 30 mg calystegine B_3_/kg d.w., while the roots in addition contained calystegine A_3_ and B_1_ [54].

The plants of the *Scopolia* spp. (Japanese belladonna) contain several TAs, mainly hyoscyamine and scopolamine, and they also contain calystegines A_3_, A_5_, B_1_, B_2_, B_3_, B_4_, C_1_ [73].

#### 2.2.4. Surveys and Reports on Associated TAs in Food in Europe

Surveys on the occurrence of TAs from associated weed plants in food mainly focus on occurrence in cereals, mainly buckwheat. Few data are available on associated TAs in other foods such as tea and vegetables.

##### TAs from Associated Weed Plants in Cereal-Based Food on the European Market

In Serbia in 2001 a case of poisoning was reported that was linked to a large consignment (about 3000 kg) of buckwheat flour imported from a neighboring country [82]. Seeds resembling those of *D. stramonium* were separated and analyzed by High-Pressure Liquid Chromatography (HPLC) with UV detection, which confirmed the presence of atropine and scopolamine.

A food poisoning incident in Slovenia in 2003 involved buckwheat that was contaminated with *Datura* [83,84]. A follow-up survey was conducted of 75 samples of buckwheat products (whole grains (N = 12), groats (N = 13), flour (N = 34), pasta (N = 8), bread (N = 4) and semi prepared ‘žganci’ meal (a breakfast dish of boiled buckwheat with oil and salt) (N = 4)) collected in Slovenia [83,84]. All 12 whole buckwheat grain samples contained seeds of *D. stramonium,* while no seeds were observed in the groats samples (N = 13). The other 50 samples of food products were analyzed by LC-MS/MS. Eleven of the thirty-four flour samples were contaminated with atropine and scopolamine at levels above the Limit of Quantification (LOQ) of 30 µg/kg, as well as four pasta samples (N = 8) and three ‘žganci’ samples (N = 4). A maximum contamination level of 26 mg/kg atropine and 12 mg/kg scopolamine was found in a sample of buckwheat flour originating from Hungary. The average TA content of the 18 positive samples was 2000 µg/kg atropine (N = 17) and 1300 µg/kg scopolamine (N = 14), with median concentrations of 330 and 190 µg/kg, respectively [84].

Atropine and scopolamine were found in buckwheat flour intended for human consumption in France in 2007 after two cases of human intoxication were reported in 2007. Twenty-six samples of buckwheat and buckwheat flour and two samples of potato pancake of various geographic regions, on the market in France in 2007, were analyzed. The sum of the atropine and scopolamine was above 100 µg/kg in 15 buckwheat or buckwheat flour samples and in the two potato pancake samples, with a maximum of 7400 µg/kg. The contamination in samples of buckwheat and buckwheat flour obtained in 2008 was much lower, as only six of thirty-four samples contained concentrations of atropine and scopolamine exceeding 100 µg/kg [85]. In 2008, a provisional threshold level of 100 µg/kg for the sum of atropine and scopolamine in buckwheat flour intended for human consumption was set in France [85].

In 2011, a limited survey on atropine and scopolamine occurrence in 16 food samples was carried out in Italy (buckwheat seeds (N = 3, whole seeds, dehulled seeds, hulls), buckwheat flour (N = 6), buckwheat pasta (N = 3), porridge (N = 2) and one sample each of crackers and flakes, sold in Italy) [86]. No TAs were detected in any of the samples above the LOQ of 1 µg/kg for atropine and 6 µg/kg for scopolamine.

In a survey conducted in the Netherlands between 2011 and 2014, TAs were quantified in 113 cereal-based foods for infants and young children [87]. TAs were only detected in cereals to be mixed with milk, with the highest concentrations linked to products containing multigrain ingredients—but no obvious ingredient was identified. The average levels were 4.6, 4.4 and 0.5 μg/kg in the cereals from 2011, 2012 and 2014, respectively, with the highest levels being 80.8, 57.6 and 3.9 μg/kg over these years. Both the average and maximum levels were clearly lower in the samples from 2014 as compared to those from 2011 and 2012, possibly due to measures taken by producers in response to the 2013 EFSA opinion on TAs. Atropine was present at higher levels than scopolamine, with relative ratios of 3.0, 4.1 and 1.7 in 2011, 2012 and 2014, respectively. The ratio observed for atropine and scopolamine was indicative for *D. stramonium* [87].

The details of the EFSA survey on the occurrence of TAs in products available at retail stores in Europe, published in 2016 [31], are summarized in Table S2. A total of 1305 samples were analyzed by LC-MS/MS for a set of 24 TAs from different structural groups: LMW tropanes, LMW tropane esters, Convolvulaceae-type TAs and *Datura*-type TAs, including atropine and scopolamine. Food categories included single-component flours, cereal-based food products and other products available at retail stores in Europe. Of all the samples, 22.5% contained one or more TAs. The mean TA concentration was 12.9 μg/kg with a range of <LOD in pasta to 130.7 μg/kg in cereal-based meals for children. The maximum total TA content ranged from <LOD in pasta to 4358 μg/kg in a sample of dry herbal tea. One or more TAs were detected in 21.3% of the single component flours (268 samples analyzed), with the highest contamination incidence (23.5%) and level (361.2 µg/kg) in sorghum and millet samples. Atropine and scopolamine were the major TAs with respect to incidence and concentration, with minor contributions of anisodamine, pseudotropine and norscopolamine. In the category ‘cereals available at retail stores’ (838 samples), 14% of the samples were contaminated with TAs. Atropine and scopolamine were the dominant TAs, while smaller contributions were made by anisodamine and pseudotropine. Substantial differences were seen between sub-categories. In the category bread, 15.8% of the samples contained TAs, but none of the pasta samples were positive. The highest mean and maximum concentrations were seen in the breakfast cereals (0.63 μg TA/kg and 111.8 μg TA/kg, respectively). TAs were detected in 20% of the cereal-based foods for young children in the age range of 6–36 months. The category ‘pasta and cereal-based meals’ (18 samples) contributed most to the contamination, both in incidence (55.6%) of the positive samples and in mean (130.7 μg TA/kg) and maximum (859.5 μg TA/kg) concentrations. The predominant TAs in these samples were tropine and pseudotropine, and these samples have been described in Section 2.1.5. Of both breakfast cereals and cookies for children, 13% of the samples were contaminated with TAs. The contamination of the samples in the categories ‘cookies for children’ and ‘biscuits and pastry’ were comparable in incidence: 13.1% and 14.6%, respectively. However, both mean and maximum concentrations were higher in the category ‘cookies for children’ (0.85 μg TA/kg and 86.2 μg TA/kg, respectively) than for the category ‘biscuits and pastry’ (0.14 μg TA/kg and 12.0 μg TA/kg, respectively). Atropine and scopolamine were the TAs most often found in these samples, but generally at low levels (max 4.2 μg/kg).

The results of a survey of TAs in twelve samples of buckwheat flour, six samples of buckwheat pasta and eight samples of buckwheat bakery in Italy were published in 2018. TAs were detected in one sample of buckwheat flour (83.9 µg atropine/kg and 10.4 µg scopolamine/kg), one sample of buckwheat pasta (21.3 µg atropine/kg and 5.7 µg scopolamine/kg) and one buckwheat bakery product (13.9 µg atropine/kg) [88].

A total of 18 cereal-based baby food samples for children under 1 year old were collected in Spanish supermarkets and analyzed for TAs in 2018. The samples were porridge (N = 7), biscuits (N = 9), one sample of a cereal snack and one breadstick sample (grissini). One biscuit sample with buckwheat as the main ingredient was contaminated with atropine at 11.5 μg/kg and scopolamine at 2.8 μg/kg; both were above the EU legal limit at the time. In addition, apoatropine was present in the sample at a level of 7.5 μg/kg, and traces of anisodamine and homatropine were detected [89].

In 2020, a total of 15 gluten-free grains and flours purchased from local shops in Madrid were analyzed for atropine and scopolamine [90]. Atropine was detected above the LOQ (1.5 µg/kg) in four of the five pseudo-cereal samples (6.7–21 µg/kg) and in four of the six cereal samples (6.9–78 µg/kg). However, scopolamine (LOQ: 2.4 µg/kg) was only detected (28 µg/kg) in the cereal sample containing the highest amount of atropine.

A total of 103 maize-containing foods (corn grit (N = 33); polenta (N = 39); semolina (N = 31)) were purchased in supermarkets in the Republic of Serbia in 2021 and analyzed for atropine and scopolamine at an LOQ of 1 μg/kg [91]. Atropine was detected in 13 corn grit samples in the range of 2.28–16.33 μg/kg. In 11 of these samples, scopolamine was detected as well (range 1.12–6.16 μg/kg). In seven polenta samples, atropine (1.10–3.98 μg/kg) was detected, while four of these samples also contained scopolamine (1.07–2.80 μg/kg). Finally, in 12 semolina samples, atropine was found (1.20–58.8 μg/kg); eight of these also contained scopolamine (1.1–10.2 μg/kg) [91].

##### TAs from Associated Weed Plants in Tea on the European Market

The plant parts of TA-containing weeds can contaminate (herbal) teas and vegetables due to the co-harvesting of the weeds or the co-mingling of raw materials.

Atropine and scopolamine levels were studied in 70 samples of dry teas (N = 10 for each of black tea, green tea, mixed herbal tea, peppermint tea, chamomile tea, rooibos tea and N = 5 for each of fennel tea and melissa tea) available on the Israeli market in 2015. The TAs were below LOQ (1 µg/kg) for black tea, green tea, mixed herbal tea, and melissa tea. One rooibos and one chamomile sample contained TAs at 2 µg/kg and one fennel sample at 94 µg/kg. The highest concentrations of TAs were detected in eight of ten samples peppermint tea, ranging from 34–379 µg/kg with a mean of 181 µg/kg [92].

From the 2016 EFSA study, in the category ‘other products’ the dry (herbal) tea samples showed a high incidence of positive samples (70% of 121 samples analyzed) (see Table S2). Atropine and/or scopolamine above the LOD (0.05–0.2 μg/kg) were found in 63.8% of the teas, with a mean and maximum concentration of 13.4 and 428.5 μg/kg, respectively. Several other TAs were found with a lower incidence in dry tea, including tropine, pseudotropine, convolidine and, notably, convolvine. Convolvine was found in 8% of the samples in concentrations ranging from 3.1 to 4070 μg/kg in a mixed herbal tea. Overall, the survey revealed that at least 19 TAs did occur in the foods analyzed [31]

A series of 11 teas, obtained from supermarkets in Spain before 2018, was analyzed for 13 TAs (of which three were grouped as physoperuvine, pseudotropine and tropine) in dry products (green tea with mint, green tea with mate, Piccadilly tea, red Chinese tea, herbal tea (Rooibos (N = 2)), *Mentha pulegium*, relaxing tea, Ayurvedic spiced tea, cocoa leaf tea and mixed tea (*Cassaia angolensis* and *Rhamnus frangula*)). The LOQs ranged from 5 μg/kg to 15 μg/kg. A total of seven TAs were detected in the cocoa leaf tea at a high concentration, ranging from 27 µg atropine/kg to 4340 µg/kg for the group of physoperuvine, pseudotropine and tropine. The group of physoperuvine, pseudotropine and tropine was present in four of the other samples ranging from 14–16 µg/kg, with co-occurring homatropine in two samples (29 and 34 µg/kg) and atropine in one sample (9 µg/kg). Apoatropine (5 µg/kg) was detected in one sample [68].

A total of forty-four herbal teas, nine herbal extracts and seven herbal tablets were purchased from the Italian market in 2019 and analyzed for atropine, scopolamine, anisodamine and homatropine at an LOQ of 0.5 µg/kg in liquid infusion, corresponding to 25 µg/kg in the dried product [93]. One herbal tea sample contained both atropine (25 ± 6 μg/kg d.w. product) and scopolamine (50 ± 25 μg/kg d.w.), while in another herbal tea sample atropine was detected (69 ± 9 µg/kg d.w.). A trace of anisodamine was detected in one sample of *Lepidium meyenii* (maca), while homatropine was not detected in any of the 60 samples.

Martinello et al. (2022) analyzed tea infusions made from 33 dry teas obtained in Italian supermarkets in 2020 for TAs [94]. Scopolamine was detected in five of the tea infusion samples in the range of 0.007 to 1.517 µg/L and atropine was detected in four of the five samples in the range of 0.011 to 0.881 µg/L. The highest level (2.4 µg/L) was detected in the tea infusion made from a herbal tea composed of licorice, rhubarb, mallow and fennel, thus exceeding seven times the EU maximum level of 0.2 µg/L [29].

##### TAs from Associated Weed Plants in Vegetables on the European Market

Castilla-Fernández et al. (2021) conducted a survey in Spain on TA contamination (LOQ of 0.015 μg/kg f.w.) in 41 frozen spinach products for adults as well as in 25 spinach-containing products for infants [95]. Atropine was detected in three of the spinach-based infant food products (range 0.02–0.06 µg/kg f.w.) and scopolamine in one (0.05 µg/kg f.w.). Three of the frozen spinach samples contained atropine (range 0.14 to 4.52 µg/kg f.w.) and thirteen samples contained scopolamine (range 0.04 to 8.19 µg/kg f.w.). The authors concluded that contamination was most likely due to *Datura innoxia* and not to *D. stramonium*, based on the relatively high proportion of scopolamine. The leaves of *D. innoxia* also show more resemblance to spinach leaves than the leaves of *D. stramonium*, making detection of the weeds in the field difficult.

##### TAs in Animal Derived Foods on the European Market

Lamp et al. (2021) conducted an animal trial in 2021 in which they studied the effects of TAs on animal health and their transfer to milk in Holstein Friesian cows [96]. Four cows received atropine and scopolamine, administered on a daily basis via capsules, in three subsequently increasing levels (93, 186 and 279 µg (sum of pharmacologically active alkaloids)/kg b.w./day), each level for 5 days. The behavior of the animals and the milk yield were not influenced by any of the dosage levels. Overall, the transfer of TAs was low, and a clear distinction was observed in transfer for the two compounds. At the highest dosage, the mean transfer rate for atropine was 0.037% and for scopolamine it was 0.007% [96]. At the highest dosage level, the TA concentration (sum of pharmacologically active alkaloids) in the milk reached 1.6 µg/kg skimmed milk.

One paper reporting on the presence of TAs in porcine muscle, chicken eggs and milk due to transfer was retrieved from the literature databases; however, it did not concern products from the European market. In 2019, Zheng et al. (2019) analyzed (-)-hyoscyamine and scopolamine in 10 samples of each of the mentioned matrices bought in the Republic of Korea [97]. None of the TAs were detected above the LOQ of 5 μg/kg scopolamine and 2 μg/kg (-)-hyoscyamine.

The presence of atropine above the LOQ (0.5 µg/kg) was observed in nine of forty Acacia and multifloral honey samples declared as of Italian or EU origin, obtained from Italian stores by Martinello et al. in 2017 [98]. Scopolamine was not detected. The concentration of atropine in five of the nine samples was above 1 µg/kg, with the highest concentration being 3.8 µg/kg [98]. On the other hand, Romera-Torres et al. (2020) reported scopolamine at 27 µg/kg, but no atropine above the LOQ (20 µg/kg), in one of nineteen samples of honey obtained from the Spanish market [99].

TAs were detected in two out of forty-seven samples of pollen obtained from beekeepers in the northern Italian regions of Veneto, Valle D’Aosta and Emilia Romagna in 2019 and 2020 [94]. Scopolamine (LOQ: 0.5 µg/kg) was detected in both samples at 4.3 and 4.2 µg/kg, respectively, and atropine (LOQ: 0.2 µg/kg) in the latter sample at 6.7 µg/kg.

#### 2.2.5. Stability of Associated TAs during Storage and Processing

Co-harvested weed parts can be cleaned from plant-based produce after harvest. However, TAs can remain in dust or be transferred via abrasion to the cereals, and they can thus contaminate the cereals even up to above the EU legal limit [100]. Transfer can already start on the inside of the harvester equipment. As illustrated in the previous paragraph, TAs have been identified in various processed foods, thus indicating at least some resistance of TAs to food processing practices.

Marin-Saez et al. (2019) studied in detail the fate of TAs during bread baking, pasta cooking and tea brewing [101,102]. Bread contaminated with TAs was prepared by spiking a buckwheat/millet dough with seeds from *D. stramonium* or *Brugmansia arborea*, followed by yeast proofing and baking [101]. The content of individual TAs (atropine, scopolamine, anisodamine, littorine, aposcopolamine, homatropine, apotropine, scopoline, tropine and tropinone) after proofing and baking was investigated. During 1 h of proofing at 37 °C, most TAs decreased in concentration, but some increases were also observed. Atropine, scopolamine, anisodamine, littorine, homatropine and scopoline decreased by 19 to 65% (average 40%). The levels of aposcopolamine, apoatropine and tropine increased, although there were differences between the *Datura* and the *Brugmansia* spiked materials. It is plausible that these TAs are formed by the degradation of the first set of TAs. After subsequent baking for 40 min at 190 °C, a further decrease (average 27%) was noted for the first set of TAs, resulting in an overall degradation by 52 to 85% (average 73%) [101]. Similarly, buckwheat that was artificially contaminated with *D. stramonium* or *B. arborea* seeds was used to prepare pasta by boiling for 10 min. A substantial reduction of TA levels in the pasta due to extraction by the boiling water (on average 30%) and degradation (on average 43%) were noted, the latter varying between 24% for apoatropine to 66% for tropine [102].

Marin-Saez et al. (2019) also prepared infusions from green tea artificially contaminated with the ground seeds of *D. stramonium* or *B. arborea* by taking 3 g of tea with 45 mL of boiling water, which was left for 5 min [102]. Both infusions and tea leave residues were analyzed. It was concluded that part of the TAs was extracted by the boiling water (between 19.5% and 94.6% depending on the TA and the seed type, with an average of 48%). A smaller part of the TAs remained in the tea leave residue (between 0 and 41.6%, on average 15%) and the remaining part (on average 24%) was lost due to decomposition or converted to other TAs [102]. The reported boiling water extraction efficiency was similar to Mulder et al. (2016) who estimated the transfer of atropine and scopolamine from contaminated dry tea into infusion at, respectively, 54 and 42% [31].

The preparation of breadsticks includes only a heating step [103]. TA degradation was studied in breadsticks made from corn flour contaminated with various levels of the ground seeds of *D. stramonium* (atropine 1911 ± 283 mg/kg) or spiked with scopolamine and anisodamine and subsequently baked for 20 min at 180 °C. After baking, the breadsticks’ level of atropine was reduced by 7–65%, and that of scopolamine and anisodamine was reduced by 35–49% [103].

#### 2.2.6. Emerging TA-Containing Weeds

Information on emerging weeds containing inherent TAs is scarce. The major plant families known to contain TAs that contaminate field crops in Europe or have a strong potential to do so were identified as Convolvulaceae and Solanaceae species with a lesser risk from *Atropa*, *Brassica* and *Datura* species. For the European situation, *A. belladonna*, *C. arvensis*, *D. stramonium* and *Hyoscyamus* are the most important TA-containing weeds with invasive potential.

*D. stramonium* is associated with the contamination of cereals at harvest [100], but the scale is difficult to address. However, *D. stramonium* was among the weeds described as a major obstacle to sunflower seed production in Turkey [67].

*H. albus* (white henbane) has been described as a natural weed in Europe [104,105,106]. *H. niger* (black henbane) is a weed of poppy, wheat and millet crops. It can produce up to 400,000 seeds per plant [107]. The literature search did not provide information on the magnitude of the associated weed problem.

*C. arvensis* (field bindweed) is considered by agriculturists and horticulturists as one of the most invasive weeds in the world [75]. It abundantly grows in temperate and Mediterranean climates where it is a weed of cereals, beans and potatoes. According to a study on the occurrence of noxious weeds in European agriculture, it is considered a problematic weed in France, Germany, Greece, the former Yugoslavia and many countries outside Europe [108]. In the same study, *C. arvensis* ranked seventh in importance with respect to other European weeds, fifteenth in the rankings for its importance in spring cereal crops and in winter rapeseed, sixth in maize and sorghum and fifth in sunflower and soybeans. Like *D. stramonium*, *C. arvensis* hinders sunflower seed production in Turkey by invading fields throughout the growing season [67].

*B. campestris* (wild turnip) is generally distributed as a weed in the temperate zones of Europe and is a cultivated plant in parts of the Middle East and Asia. It is found throughout the western part of Russia, in the Caucasus and in Western and Eastern Siberia. It has been described as a pernicious weed infesting all spring crops, both grain and vegetative types [107].

A number of other Brassicas that are mentioned as weeds and that contain calystegines are probably of lesser concern: *Arabidopsis thaliana*, *Coringia orientalis* (toxic to livestock, noxious weed), *Cronopus squamatus*, *Diplotaxis murales*, *Isatis tinctoria*, *Lunaria annua* (not considered to have weed potential), *Lunaria rediviva* (not considered to have weed potential), *Matthiola incana* (not considered to have weed potential), *Neslia paniculata* (commonly affecting wheat and millet), *Peltaria alliacea* and *Sisymbrium strictissimum* [107].

### 2.3. Notifications on TAs in the European Union RASFF

A search of the RASFF, updated on 31 December 2022 [109], produced 83 relevant results from the search terms: atropine, scopolamine, *Atropa*, *Belladonna*, *Datura*, *Hyoscyamus*, *Mandragora*, and *Solanum*. No results were obtained for the names of other TAs or other TA-producing plants. The results are described below in alphabetic order (Section 2.3.1, Section 2.3.2 and Section 2.3.3).

All results for scopolamine were also returned under the atropine search with a total of 58 alerts and notifications between 1994 and 31 December 2022 (see Table S3). The EU RASFF at that date contained a total of 25 notifications (information and alerts) for *Atropa, Belladonna*, *Datura*, *Hyoscyamus*, *Mandragora*, and *Solanum* (see Table S4). The data of the EU RASFF notifications (alerts and information) are summarized in Table 3.

#### 2.3.1. Atropine and Scopolamine

A total of 58 notifications and alerts were reported for atropine and scopolamine in the period 1994 to 31 December 2022. The reports were on seven food product categories and the category feed. A total of thirty-nine reports were from the product category ‘cereals and cereal-based products’, seven reports from ‘cocoa and cocoa preparations, coffee and tea’, five reports from ‘dietetic foods, food supplements, fortified foods’, four reports from ‘herbs and spices’ and one report each from the categories ‘food additives and flavorings’, ‘fruits and vegetables’ and ‘nuts, nut products and seeds’. For 28 of the 39 reports in the product category ‘cereals and cereal-based products’, TA concentrations were reported for the sum of atropine and scopolamine, which ranged from 4.0 to 1014 µg/kg. A maximum of 20,835 µg/kg of the sum of atropine and scopolamine was reported in 2018 for whole cumin seeds (RASFF 2018.0774). Most reports within the product category ‘cereals and cereal-based foods’ were on ‘corn-based food, popcorn’ (13 reports), on ‘millet and millet-based food’ (11 reports) and on ‘buckwheat’ (9 reports). From some incidents, multiple RASFF reports were filed by various EU countries. The results indicate that TA-containing weeds occur in many fields on which cereals are grown. After harvest, the weeds (seeds) are not fully removed and are ground in the flour for food production.

#### 2.3.2. Datura

In the period 2006–31 December 2022, there were 16 notifications and alerts for Datura. Five notifications of *D. stramonium* seeds in food were related to millet and one to oatmeal. Four alerts were on the occurrence of *Datura* seeds in feed. *Datura* seeds were detected in three vegetable mixes: one bacon stir-fry mix from Spain in 2007 (RASFF 2007.CGO), one frozen vegetable-bean-seed mix from Belgium/Spain (RASFF 2013.0696) and one on frozen beans from France in 2019 (RASFF 2019.0993). *D. stramonium* fruits (presumably unripe seed cases) were found in canned green beans from Hungary in 2006 and in 2007 (RASFF 2006.0835 and RASFF 2007.0613) and in canned peeled tomatoes in 2019 (RASFF 2019.3340). The notifications show that the co-harvested weeds are present as seeds in the products and can be recognized easily in whole products. The presence of the fruits of *Datura* can cause a high concentration of TAs in the products from the product category ‘fruits and vegetables’. This was shown in the notification alert on frozen spinach puree (RASFF 2021.1390) where the concentration of atropine ranged from 850 µg/kg to 3466 µg/kg and that of scopolamine ranged from 1033 µg/kg to 3860 µg/kg. This case shows that in processed foods, TAs should be identified by chemical analysis.

#### 2.3.3. Hyoscyamus, Mandragora, and Solanum 

Seeds of *H. niger* (henbane) were found as contaminants in poppy seeds from the Czech Republic in 2007 and 2008 (RASFF 2008.0520; RASFF 2007.0267; RASFF 2007.0256). The contamination level was between 0.13% and 0.42%. Two of the samples were sold in Slovakia and the third in the Czech Republic.

In October 2022, leaves of mandrake leaves were detected in fresh spinach produce in Italy (RASFF 2022.5877) after reported cases of illness. The product was withdrawn from the market. 

*S. niger* was detected in frozen peas originating from Belgium in 2021 (RASFF 2021.7140). The product was withdrawn from the market.

### 2.4. Reported Cases of Human Intoxication in Europe

Reports of human intoxications in Europe following everyday food consumption or due to the misidentification of collected wild berries are rare. Some of them are described in relation to *A. belladonna* (Section 2.4.1), *Datura* (Section 2.4.2) and other TA-containing plants (Section 2.4.3), as well as reported by poisoning centers (Section 2.4.4). Results from the reports are summarized in Table 4. On the other hand, many reports can be found on the acute adverse effects of TAs in humans, resulting from the deliberate ingestion of TA-containing plant parts in order to experience hallucinogenic effects [110]. The latter will not be discussed in this paper.

#### 2.4.1. Atropa Belladonna-Human Intoxications in Europe

Accidental intoxications of humans (adults and children) by TAs from *A. belladonna* result from the mistaken identification of the berries as edible berries (such as bilberries, blueberries, cranberries and huckleberries) and when roots or leaves have accidentally been used for making tea or mistakenly used as a vegetable [1,26]. It was estimated that a lethal dose for children may be reached when ingesting 2–5 berries of *A. belladonna* and for adults 10–20 berries [26].

Several cases were found for the mistaken identity of the berries of *A. belladonna*. An elderly but healthy man in Italy was accidentally poisoned with *A. belladonna* berries [111]. The serious poisoning of a Danish 9-year-old boy resulted from the ingestion of 20–25 *A. belladonna* berries [112]. In both cases the symptoms were mistaken for other diseases, namely acute onset of senile dementia for the Italian patient [111] and acute psychosis for the Danish boy [112].

A 52-year-old female in the Netherlands suffered from an anticholinergic syndrome after the ingestion of berries from *A. belladonna* mistaken for bilberries [113]. The case report of an otherwise healthy 48-year-old adult male in Germany described severe adverse health effects after ingesting three handfuls of berries from *A. belladonna* [114].

A report from 1996 describes the intoxication of four adults and four children in France who accidentally picked ripened *A. belladonna* berries instead of whortleberries [115]. All consumed 3–6 raw berries on the day of collecting the berries, after which they all experienced mild symptoms that remained unnoticed. The berries were cooked in a pie the next day and consumed by three of the adults. Within 2 h, the three adults experienced severe symptoms such as becoming delirious with visual hallucinations; one of them became comatose and had to be hospitalized [115]. Atropine was detected in the urine of all three adults.

TAs in tea caused poisoning in Austrian consumers after drinking stinging nettle tea (*Urtica*) contaminated with leaves of ‘belladonna’ [1,116].

Comfrey tea accidentally contaminated with *A. belladonna* leaves led to poisoning incidents in the UK in 1983 when an elderly couple suffered from atropine-like symptoms after drinking comfrey tea to relieve rheumatic symptoms [117,119]. Related to the same case of contaminated tea, a 30-year-old male suffered from atropine-like symptoms after drinking comfrey tea (*Symphytum*) to relieve flatulence. The tea leaves contained 4 mg atropine per 28 g [118,119].

A single case of poisoning by a herbal tea (described as a ‘biodrug’) in Germany causing ‘respiratory insufficiency’ was reported by a physician in 2001 [18]. The intoxication was linked to *A. belladonna* [18].

Lungwort tea (*Pulmonaria officinalis*) accidentally contaminated supposedly with *A. belladonna* led to the severe intoxication of three members of a Spanish family—a male 76 years of age and females 42 and 14 years of age—when drinking the tea to relieve the common cold [119,120].

In 2013, eight adults were poisoned in the Netherlands in at least two incidents involving the consumption of herbal tea prepared from approximately 20 g of what was assumed to be dried marshmallow root (*Althaea officinalis*) [20,121]. A poisoning case linked to the same product was reported in France after the purchase of the herbal tea in the Netherlands. For two adults, it was confirmed that they had been exposed to a dose of 20 to 200 mg of atropine, 10–100 times the dose that can cause symptoms [121]. The tea contained a high content of atropine (1–10 mg/g), and *A. belladonna* (deadly nightshade) was identified as the probable source [20,119]. The alleged *Althaea officinalis* was harvested in Bulgaria and sold only in the Netherlands. The patients had anticholinergic symptoms (dry mucous membranes, nausea, blurred vision, hallucinations, tachycardia and urinary retention) typical of tropane poisoning [20].

#### 2.4.2. Datura-Human Intoxications in Europe

Reports on intoxications of humans by *Datura* have resulted from co-harvested *Datura* plant parts causing elevated TA levels in cereals (flours) or vegetables mixtures, or by mixing leaf vegetables. *Datura* is an invasive plant that appears to readily contaminate buckwheat (*Fagopyrum esculentum*).

In 2012, a stock of Breton buckwheat purchased by a mill in the Alpes de Haute Provence caused food poisoning in a large area of southeastern France [19]. The flour from this stock was sold in over 100 bakeries, retail outlets, organic shops and restaurants. Within 2 months, 24 people in the Provence-Alpes-Cotes d’Azur and Languedoc-Roussillon regions presented to accident and emergency departments with symptoms of *Datura* intoxication. This was confirmed in 19 of these patients from 12 households. The levels of atropine and scopolamine in the flour of the mill from the Provençal region were much higher (16,467 and 7042 µg/kg respectively) than in mills from different parts of France. In the Rhone-Alpes region, fourteen people from nine different households were intoxicated most probably from the same batch of buckwheat. After the product was withdrawn from the market, cases of poisoning continued to be recorded from places in Rhône-Alpes; one case was even reported after 5 months [19].

In Slovenia in 2003, 73 cases of domestic food poisoning with mild to moderate effects were associated with the ingestion of a traditional dish containing buckwheat flour [17]. The flour for the dish was prepared from whole buckwheat grain which contained up to 190 seeds of *D. stramonium* per kg of grain. Atropine and/or scopolamine were detected (>3 µg/kg) in 20 of the 43 buckwheat flour samples provided by consumers of dishes containing buckwheat. The highest levels, 26 mg/kg atropine and 12 mg/kg scopolamine, were found in flour consumed by a family of eight people. It was calculated that the intake by the members of this family had been between 53 and 140 µg/kg b.w. for atropine and between 25 and 64 µg/kg b.w. for scopolamine. At the time, all buckwheat flour and buckwheat flour products were recalled in Slovenia, and the national legislation on grain purity and buckwheat flour was amended following a risk-assessment study [17,84].

In 2007, two cases of human intoxications caused by TAs in buckwheat pancakes were filed in France [85]. Details on the cases are not available, but the cases were used to determine an intervention threshold of 100 µg/kg for the sum of atropine and scopolamine in buckwheat flour intended for human consumption [85].

*D. stramonium* seeds present in millet-carrot balls in Austria caused illness in eight persons, of which one had to be hospitalized [23]. The batch of millet used to prepare the millet-carrot balls was contaminated with 50 seeds of *D. stramonium*/kg. It was estimated that each person consumed three seeds of *D. stramonium*.

In 2010, a batch of canned beans contaminated with *D. stramonium* was reported to have caused three poisonings in France [122]. The product was recalled from the market.

Frozen mixed vegetables contaminated with seeds of *D. stramonium* were sold via the largest market chain in Finland in May of 2013 [21,126]. A total of 28 poisonings were suspected, of which 10 patients were confirmed with anticholinergic symptoms. The products were recalled, and 30,000 customers were warned to avoid the vegetables [21].

In the spring of 2021, about 100 persons filed complaints at the National Toxicological Information Center in Bratislava in the Slovak Republic after the consumption of spinach prepared from frozen spinach (RASFF 2021.1390 [109,123]). The frozen spinach product was also available from the Czech Republic market. TAs were detected in high concentrations in the frozen spinach products, and the products were recalled in both countries [123]. The samples contained atropine between 850 and 3446 µg/kg and scopolamine between 1033 and 3860 µg/kg [109].

A 10-year-old girl in Germany suffered from *D. stramonium* intoxication after accidentally drinking tea prepared from dried *D. stramonium* leaves [124]. The leaves were intended for the production of vapors to be inhaled for the treatment of asthma.

A 53-year-old female suffered from TA poisoning after consuming homemade pumpkin blossom fritters, to which she added some leaves from a large shrub in her garden later identified as *D. stramonium* [24].

In Greece, seven people were poisoned by the consumption of cooked leaves of *Amaranthus blitum* (blites) that were contaminated with leaves of *D. innoxia* [125]. The leaves were collected in the wild. The left-over cooked vegetables were examined for atropine and scopolamine and levels of 0.8 and 1.2 mg/kg, respectively, were found. The patients recovered with hospital treatment.

#### 2.4.3. Other TA-Containing Plants-Human Intoxications in Europe

A case of misidentification was responsible for the TA intoxication of two consumers in Crete, Greece. What was thought to be the leaves of *Borago officinalis* turned out to be *Mandragora officinarum* leaves, known to contain TAs, although this was not confirmed by chemical analysis [22]. After hospitalization for one week, the patients had recovered.

In Spain, 15 persons suffered from intoxication by *M. autumnalis* that was intermingled with chard and spinach leaves. Hyoscyamine and atropine were identified in the product consumed [127].

In October 2022, at least 12 persons were intoxicated after the consumption of fresh spinach produced in the Abruzzo region in Italy (RASFF 2022.5877 [80,81,109]). Based on the reported symptoms, mandrake (*M. autumnalis*) was implicated as the most likely source of contamination. The resemblance of mandrake leaves with that of spinach was noted.

#### 2.4.4. Poisoning Center Reports in Europe

Plant exposures in children was the fourth-most common cause (22% of the exposures) for calling the Poison Information Centers in Germany in the period 1998–2004 [128]. Analysis of 58,641 case reports from the mentioned time period revealed that at the moment of calling, 9.6% of the children showed clinical effects after exposure, with 0.4% of the children showing moderate to major effects [128]. *Datura* was among the plants causing the most severe poisoning [128].

Information on severe poisonings by plants (135 case reports analyzed) reported to the Swiss Toxicological Information Center from 1966 to 1994 showed that severe poisoning was associated with 24 different plant species, including the TA-containing plants *A. belladonna* (42 cases), *D. stramonium* (17 cases) and *H. niger* (3 cases) [129].

From 174 exposures of children to plants recorded in the Czech Republic over a 6-year period from 1996 to 2001, the most frequent cases (15%) resulted from the ingestion of *Datura* seeds [130].

### 2.5. Factors Influencing Exposure

#### 2.5.1. Plant Cultivar, Agricultural Management and Climate

Plants that produce inherent TAs—food crops as well as weeds—in general produce a profile of TAs that is correlated to the genetic make-up of the plant family, geographic origin, growth state and environmental factors such as climate or handling of the product [5,6,7,8,9].

The calystegine content was studied in eight potato cultivars by Friedman et al. (2003) [35]. They reported that the sum of calystegine A_3_ and B_2_ in the wet flesh differed 60 times between the lowest and highest value within the eight cultivars [35]. The study by Petersson et al. (2013) confirmed the cultivar dependency of calystegine presence and concluded that the highest variation was observed in calystegine B_4_ levels [11]. The wounding of the potato tubers or exposing the tubers to light did not influence the calystegine concentrations [11]. The dependence of the profile and concentration on the cultivar was also detected in tomato, as described by Romera-Torres et al. (2019) [9]. Similar findings have been observed for the other TAs occurring in weed plants, e.g., for the plants in the *Datura* genus [63] and the Convolvulaceae family [7]. It was shown that genera within the family of Convolvulaceae can be distinguished based on their chemo-taxonomic profile of calystegines.

Agricultural management or changing climate conditions may cause weeds to spread or become invasive. Besides the occurrence and number of plants, climate change can also cause weed plants such as *D. stramonium* to grow larger and produce more seeds, even at water-limiting conditions [131]. In the present study, no papers were identified describing the effects of changes in agricultural management and growing conditions on the content and patterns of calystegines in food plants or the other TAs in weed plants.

Food for the European market is sourced globally. Toxic plant metabolites from a wide variety of weeds co-harvested with the food crops, can be expected to occur in (raw) food materials imported to Europe. For example, *S. ptycanthum* is a noxious weed in fields where soybeans are grown, and the toxic metabolites of this weed can be detected in harvested food crops [132].

One way of reducing weeds in the fields is the use of herbicides. Glyphosate is most often applied [133], but it was established in 2018 that at least 38 weeds growing in all regions of the world had become resistant to this herbicide [133]. Periods of drought or increased CO_2_ concentrations also have a negative influence on the effectiveness of herbicides [134]. The trend for organic food further reduces the use of herbicides, which will most probably increase the risk of toxic weeds growing in the food crop. Therefore, the prevention of invasive weeds entering a geographic area and weed control are necessary, but this brings many difficulties [133].

After harvest, the cleaning of the crops is the next step in preventing plant parts of toxic weeds from entering the food chain. Abia et al. (2020) described in detail how contamination with seeds and other plant parts of *D. stramonium* can be avoided by applying Good Agricultural Practices (GAP) and cleaning produce by Good Manufacturing Practices (GMP) [135]. HACCP systems should be in place to evaluate and monitor the risks and avoid the mix up of raw materials, such as what happened in the Netherlands with *Althaea officinalis* tea accidently being exchanged with *A. belladonna* [20].

#### 2.5.2. Consumption Pattern

Resulting from globalization and societal challenges, diets are changing, new products are being introduced in Europe, the quantity of a product in the diet is increasing or some products are consumed by a particular group of the population.

Consumers worldwide are encouraged to increase the consumption of vegetables and fruits to support a healthy diet [136]. This may encourage consumers in Europe to eat more year-round readily available vegetables such as eggplant and bell pepper. These plants bear the risk of containing various inherent TAs, as illustrated in Section 2.1., although concentrations and patterns occurring are poorly known.

Along the line of healthy diets, plants with presumed positive health aspects can count on attention in Europe. Goji berries as such (dried) or as an ingredient in muesli, energy bars or drinks, were introduced to Europe for their presumed health aspects [41]. This plant, belonging to the Solanaceae family, may contain inherent TAs, as explained in Section 2.1.4. This may result in increased TA exposure to the consumers.

Foraging for wild edible plants (WEPs) is a habit becoming increasingly popular in Europe [137]. Increased exposure to TAs may result from this habit because of the risk of mistaken identity when foraging for berries or leaves. Intoxications from the accidental consumption of TA-containing berries or leaves have extensively been described in Section 2.4.

Regarding exposure to TAs, many reports were identified on TA co-contamination in buckwheat. Buckwheat is a gluten-free pseudo-cereal, replacing cereals in the diet of consumers with a gluten intolerance [88]. Buckwheat is also considered to have enhanced nutritional properties and therefore is increasingly used in so-called multi-cereal products [138]. As mentioned in Section 2.2., several of the products prone to contamination with TAs are specifically intended for babies and young children, such as cereals, cookies and tea [31].

#### 2.5.3. Analytical Challenges

A prerequisite for the collection of occurrence data is that validated analytical methods are developed that show good sensitivity and selectivity and use high-quality analytical standards and (certified) reference materials. Due to the global sourcing of food raw materials and climate challenges, TAs from weeds unknown to the region might contaminate the food, or unusually high levels may occur. This means that a wide variety of analytical standards must be incorporated in the methods to analyze for these emerging TAs.

Good-quality analytical standards are still lacking for many TAs. An extensive search for commercially available TA analytical standards was carried out for the 2016 EFSA study. A set of twenty-four TAs and six calystegines was obtained with the appropriate quality, and these were used to analyze all food samples [31]. Not many new analytical standards have become available since then. Isotopically labeled internal standards are available for only a few TAs, primarily for atropine and scopolamine.

Analytical methods for the quantification of TAs are nowadays mainly based on LC-MS/MS and LC-HRMS methods [31,139,140]. Low LOQs can be achieved, typically in the (sub-)µg/kg range. The extraction and clean-up of the extracts remains challenging due to the wide variety of food matrices that are relevant for TA analysis [97,140]. The methods must consider high concentrations of inherent TAs, such as the calystegines which are present at mg/kg, but also very low concentrations at µg/kg in the case of TAs originating from co-harvested weeds. A further challenge is the highly polar nature of the calystegines, for which relatively few methods, most of them using LC-MS or GC-MS, have been described [9,11,31].

## 3. Challenges on Exposure of the European Population to TAs via Food

The European population is exposed via food to TAs inherent to the foods and TAs from co-harvested TA-containing weeds. In 2016 it was observed that about 22.5% of the food on the market in Europe was found to contain various TAs, mainly from *Datura*, *A. belladonna* and Convolvulaceae weeds [31].

Cases on human intoxications due to TAs after the accidental co-mingling of raw materials for food supplements is illustrated in Section 2.4. Monitoring data on the occurrence of TAs in food supplements in Europe are largely missing, although multi-methods for the analysis of TAs have been published [139]. On the contrary, pyrrolizidine alkaloids (PAs) are regularly monitored and detected in food supplements in Europe [141]. This may indicate that co-mingling occurs more often, and the presence of TAs can also be expected in food supplements.

Few studies were found on the transfer of TAs from feed to meat, milk and eggs, which seems to be limited based on the information available [96]. Studies on the transfer of other plant toxins such as pyrrolizidine alkaloids and quinolizidine alkaloids show that when the plant toxins are present in animal feed via roughage, they can be transferred to milk and eggs, although the rates vary significantly [142,143,144,145]. The rate of transfer is probably correlated to the extent that degradation and metabolism occurs in the gastro-intestinal tract and the liver of animals [143]. Besides lack of occurrence data in animal-derived foods, data on the occurrence of TAs in animal feed including roughage are hardly present. One paper was retrieved on the occurrence of TAs in animal feed in Spain, indicating the presence of TAs in 40% of the samples ranging from 5 to 25 µg/kg [146].

In this paper so far, the exposure of the European population via consumption of foods has been evaluated. The scope might be expanded when considering drinking water. It is known that toxic compounds from crop plants, ornamental plants and weeds can leach via the soil to sources of drinking water [147]. Animals and humans can be exposed to these toxins at water consumption. Much work has been carried out on ptaquiloside, a genotoxic sesquiterpene glycoside, which was detected in receiving waters in Ireland surrounded with extensive populations of bracken ferns [147]. Griffiths et al. (2021) exposed the water organism *Daphnia magna* to quinolizidine and pyrrolizidine alkaloids produced by lupin and *Senecio jacobaea*, respectively, which are considered as weeds in Denmark [148]. The authors observed toxic effects and concluded that natural toxins occurring from weeds should be included in environmental risk assessment [148]. TAs have not yet been considered in this respect.

In this study, foods containing the inherent calystegines were identified as an emerging issue. *S. tuberosum* (potato), *S. melongena* (eggplant), *C. annuum* (bell pepper) and *B. oleracea* (broccoli, Brussels sprouts) were identified as the food groups most relevant for the exposure to inherent TAs in food in Europe. Other common food products, however, with little evidence of inherent calystegines were *I. batatas* (sweet potato) and food plants from the Brassica family such as *B. oleracea* (broccoli, cauliflower, kale, kohlrabi and Brussels sprouts).

In the 2016 EFSA study, as described in Section 2.1.5, for the first time, LMW tropanes and Convolvulaceae-type TAs were identified as inherent TAs to *C. annuum* (bell pepper), and thus to ‘stir-fry mixes’ and ‘ready-to-eat meals for children’ containing bell pepper [31]. This suggests that exposure to LMW tropanes and Convolvulaceae-type TAs can come from both inherent TAs as well as from co-harvested weeds. Overall, it means that more data must be collected on the occurrence of inherent TAs in foods.

The TA profile, as for other inherent plant toxins such as quinolizidine alkaloids and glycoalkaloids, can depend on cultivar, climate and agricultural management. It is advised to monitor the development of TA profiles and concentrations in food plants resulting from plant breeding practices. Lessons can be learned from potato breeding, where the introduction of a new cultivar led to intoxications due to high glycoalkaloid contents [149]. Plants often produce toxins as a defense mechanism against insect attack, animal grazing or diseases. Therefore, unexpected changes in TA concentrations can occur due to plant stress caused by, e.g., climate change.

This study also showed that many relevant foods are prone to contamination with associated TAs from weeds. It is assumed that weeds will grow more regularly in crop fields since the use of herbicides is strongly discouraged [23]. In Europe, currently, this issue mainly concerns cereals, single ingredient or multi-cereal products, and millet and millet-based products, followed by buckwheat. Since infants and young children consume a relatively large amount of cereals based on their body weight, the quality of cereals intended for infants and young children is of the highest importance. Herbal teas are the second important food of foods at risk for co-contamination with TAs either by the accidental co-harvesting of weeds or due to the (un)intended co-mingling of ingredients. Tea specifically for infants and young children should receive extra attention. RASFF notifications show that vegetables available at retail stores, either canned or frozen, can be contaminated with seeds or plant parts of *Datura* or *A. belladonna*. This shows that contaminants inherent to weeds, such as TAs, can easily enter the food chain resulting from changes in agricultural management. On top of EU-produced food, food raw materials as well as feed and feed raw materials are globally sourced; TAs from weeds unknown to the European situation can also enter the food chain. This illustrates the need for food business operators to increase their knowledge of risks related to TAs when sourcing raw materials and of the implementation of well-designed HACCP systems.

An emerging risk are the TAs from bindweed, a species expected to severely contaminate crop fields in future [75]. The literature indicates the presence of calystegines in this plant family, but no papers were retrieved on the occurrence of co-occurring calystegines from these weeds. However, the TA convolvine, occurring in Convolvulaceae weeds, tropine and pseudotropine, were regularly detected in tea samples analyzed in the EFSA study [31]. This illustrates the need for effective weed management by farmers. Authorities should be involved to prevent the spreading of noxious weeds and develop mitigation strategies.

Trends in food consumption have been identified as emerging issues, but the trend to source food from nature or one’s own garden needs attention too. *Mandragora* and *Datura* were involved in intoxications when leaves were mistaken for spinach, and berries of *A. belladonna* were mistaken for edible berries. Attention must be paid to highlight the risks and to educate persons that source food from the wild.

The EU has now harmonized the regulation of atropine and scopolamine in foods, and contamination levels are now monitored by the Member State Food Safety Authorities [28]. Data on the occurrence of other emerging TAs are, however, lacking. Food Safety Authorities in Europe are advised to start collecting data on the occurrence of calystegines, LMW tropanes, and Convolvulaceae-type TAs in food and feed over an extended period of time to even out yearly variations.

## 4. Future Perspectives

The increased number of RASFF notifications related to TAs in food in recent years shows an increased awareness by farmers, food business operators and regulators of the risk of contamination of food products by TA-containing plants. The regulatory limits that have been established in the EU for atropine and scopolamine for a number of food commodities have already proven to be an effective measure to reduce the presence of these toxins in the products covered. It can be expected that the scope of the regulation will be expanded in the future when more food groups are identified by food safety authorities as being at risk for contamination by TAs. TAs other than atropine and scopolamine may in the future be included in legislation when occurrence data becomes available that confirms their presence in food.

A better understanding of the occurrence, invasiveness and preferred growing conditions of TA-containing weeds may help farmers to better identify the most important sources of contamination and enable a more effective control of the weeds at the field level. Developments in the area of on-site monitoring of weeds in crops using spectroscopic identification by imaging techniques (e.g., by using drones) and the use of artificial intelligence (AI) to identify weed plants may help farmers to locate hotspots of contamination more effectively and to remove the weeds before the crops are harvested. The development of improved mechanical weed control systems will contribute to this as well. Artificial intelligence systems will enable food business operators to identify geographic regions and crops prone to contamination with TA-containing weeds when sourcing raw materials. These technological developments will all help to counterbalance the impact of the changes in the climate and globalization and the reduced use of plant protection products, combined with the urge to increase biodiversity—factors which potentially will increase food safety risks by TAs and presumably other plant toxins.

Understanding the production of inherent TAs at a genetic and metabolomic level by food plants will provide options to control levels in the foods in the future. Plant breeding programs should therefore evaluate the genetic make-up to produce TAs, in particular for food crops such as potato and eggplant. A better understanding of the impact of climate and growing conditions on the production of inherent TAs by the food plants will lead to improved agricultural management systems by farmers. It will help food business operators to upgrade their food safety system (HACCP based) by assessing the risks of increased TA levels when sourcing raw materials. In addition, food business operators can invest in factors that reduce the levels of inherent TAs during food processing. 

Awareness of the potential presence of co-harvested weeds and good manufacturing practices among all actors in the food supply chain will reduce the risks of mistaken identity of raw materials or the mixing of toxic and non-toxic plants. The increased number of RASFF notifications on toxic plant parts in fresh or frozen leafy vegetables will encourage food business operators to improve the cleaning procedure of the vegetables. 

Analytical techniques such as LC-HRMS, are increasingly being used for the comprehensive screening and assessment of the chemical content of plants and foods. These techniques provide the tools to improve our understanding of the occurrence, diversity and prevalence of TAs from weed plants as well as on inherent TAs in food plants. The number of available TA analytical standards is still a bit limited. It may be expected that this will improve when interest in TAs continues to increase. Targeted methods of analysis in the area of official control are now well established, and analytical standards are available as well as proficiency testing schemes. For food business operators, screening tests for TAs may become available in the future as a more cost-effective way to quickly assess the quality of their food ingredients and the risk of contamination by TAs.

To allow elaborate risk assessment studies, many TAs still need to be evaluated for toxicity. This may be facilitated by the development and use of (panels of) in vitro assays and also by the application of in silico toxicology to predict toxicity.

## 5. Conclusions

This study shows that food on the market in Europe can contain inherent and co-harvested TAs, e.g., potatoes, bell pepper, eggplants, cereal-based foods, teas and foods intended for infants and young children. Changes in the occurrence of TAs in any of the food commodities due to agricultural practices, climate or food consumption trends will contribute to the exposure to the consumers. The lack of knowledge on the occurrence of most of the TAs currently prohibits a proper risk assessment. Food Safety Authorities are advised to expand the future monitoring programs with calystegines, LMW tropanes and Convolvulaceae-type TAs. Farmers, plant breeders and food business operators are encouraged to work with food safety authorities to prevent TAs from weeds entering the food chain and to mitigate the levels of inherent TAs in foods.

## 6. Methodology

This literature review focuses on the occurrence of TAs in food plants and in co-occurring weeds that contain TAs and have the potential to contaminate food in Europe. This review started from the literature search performed by Fera Information Centre (UK) in March 2015 as presented in the EFSA study [31]. Peer-reviewed papers published on the subject were collected from publicly available scientific databases at Wageningen University and Research since then. Only papers related to food or on weeds related to food were selected for reviewing. All papers were screened for information on the TAs occurring and concentration present. Reports on the TA contamination of food supplements or cases of recreational use were excluded from the study. The European Union Rapid Alert System for Food and Feed (RASFF) [109] was searched for notifications on TAs or the presence of TA-producing plants up to 31 December 2022.

## Figures and Tables

**Table 1:** Food plants containing inherent tropane alkaloids.

Family/Genus	Species	Common Name	Tropane Alkaloid(s) Reported in the Species		Reference
			Atropine	Calystegines (Country of Origin if Known: Tropane Alkaloids)	
Brassicaeae					
*Brassica*	*B. nigra*	Black mustard	NR *	A_3_; A_5_; B_2_	[32]
	*B. oleracea*	Broccoli, cauliflower, kale, Brussels sprouts	NR	A_3_; A_5_; B_2_	[32]
*Crambe*	*C. maritima*	Sea kale	NR	A_3_; A_5_; B_3_	[32]
*Lepidium*	*L. sativum*	Garden cress	NR	A_3_; A_5_	[32]
Convolvulaceae					
*Ipomoea*	*I. aquatica*	Water spinach	NR	Thailand: A_3_; B_1_; B_2_; B_4_	[7]
	*I. batatas*	Sweet potato	NR	Japan: B_1_; B_2_; C_1_ UK ^1^: A_3_; B_1_; B_2_; C_1_ Mexico, Panama, Japan: A_3_; B_1_; B_2_	[5,7]
Moraceae					
*Morus*	*M. alba*	White mulberry	NR	Japan: B_2_	[5,33]
Solanaceae					
*Capsicum*	*C. annuum var. angulosum*	Bell pepper	NR	Japan: B_1_; B_2_; C_1_	[5]
	*C. frutescens*	Chili peppers	NR	UK: A_3_; B_2_	[5]
*Lycium*	*L. barbarum* and *L. chinense*	Goji berry	Atropine	NR	[34]
	*L. europaeum*	Goji berry	Atropine	NR	[34]
*Physalis*	*P. peruviana*	Cape gooseberry	NR	UK: A_3_; B_1_; B_2_; C_1_	[5]
*Solanum*	*S. betaceum* (syn. *Cyphomandra betaceae)*	Tomatillo	NR	UK: B_2_	[5]
	*S. esculentum* (syn. *Lycopersicum esculentum)*	Tomato	NR	UK: A_3_; B_2_ Spain: A_3_; A_5_; B_2_; C_1_	[5,9]
	*S. melanocerasum* (syn. *S. Scabrum)*	Garden huckleberry	NR	UK: B_2_	[5]
	*S. melongena*	Eggplant or aubergine	NR	Japan: B_1_; B_2_ UK: A_3_; B_2_	[5]
	*S. tuberosum*	Potato (fresh tuber flesh)	NR	Japan: B_2_ Sweden: A_3_; B_2_; B_4_ UK: A_3_; B_2_ USA ^2^: A_3_; B_2_ UK: A_3_; B_2_ (French fries for microwave cooking, oven-ready chips, instant mashed potato granules, potato waffles, potato crisps, crisps from freeze-dried potato granules, hula hoops)	[5,11,35,36]

* NR = no reports found. ^1^ UK = United Kingdom. ^2^ USA = United States of America.

**Table 2:** Associated tropane alkaloids containing weed plants in food in Europe.

Family/ Genus	Species (Invasive Weed in Europe ^1^)	Common Name	Tropane Alkaloids Reported in the Species		Reference
			Tropane Alkaloids Except Calystegines	Caly-Stegines Reported	
Brassicaceae					
*Brassica*	*B. campestris* (M)	Wild turnip	NR *	A_3_; A_5_	[32]
*Camelina*	*C. sativa* (U)	False flax	NR	A_3_; A_5_; B_2_; B_3_	[32]
*Cochlearia*	*C.* spp. (L)	Scurvy grass	Tropine, pseudotropine	A_3_; A_5_; B_2_; B_3_	[32]
Convolvulaceae					
*Convolvulus*	*C. arvensis* (H)	Field bindweed	Tropine, pseudotropine, tropinone	A_3_; A_5_; B_1_; B_2_; B_3_; B_4_	[7,52]
Solanaceae					
*Atropa*	*A. belladonna *(L)	Deadly nightshade	Atropine, scopolamine, apoatropine, norhyoscyamine, hyoscyamine, 6β-hydroxyhyoscyamine, aposcopolamine, littorine, tigloyltropeine, tigloyloxytropane, tropine, anisodamine	A_3_; B_1_; B_2_; B_3_	[4,10,14,53,54,55,56,57,58,59,60,61]
*Datura*	*D. stramonium* (H)	Jimson weed, Devil’s snare	Scopolamine, atropine, hyoscyamine, 6β-hydroxyhyoscyamine, apoatropine, 3α-phenylacetoxytropane, 3-hydroxy-6-isobutyryloxytropane, 3-hydroxy-6-(2-methylbutyryloxy)-tropane, 3-(2-methylbutyryloxy)-6-hydroxytropane, 3-(3′-acetoxytropoyloxy)-tropane, 3α-hydroxy-6β-tigloyloxytropane, littorine, aponorscopolamine, aposcopolamine, 3-tigloyloxy-6-propionyloxy-7-hydroxytropane	NR	[4,12,62,63,64,65,66,67,68]
*Duboisia*	*D. leichhardtii* (L)	Not known	(-)-hyoscyamine, scopolamine	B_1_; B_2_; B_4_; C_1_; C_2_	[2,13,56,69]
	*D. myrporoïdes* (L)	Corkwood	(-)-hyoscyamine, scopolamine	NR	[56]
*Hyoscyamus*	*H. albus* (H)	Henbane	(-)-hyoscyamine, scopolamine	A_3_; B_1_; B_2_; B_3_	[54,56]
	*H. niger* (H)	Black henbane	(-)-hyoscyamine, scopolamine	A_3_; A_5_; A_6_; B_1_; B_2_; B_3_; N_1_	[2,56,70]
*Mandragora*	*M. autumnalis* (L)	Autumn mandrake	Hyoscyamine, hyoscine, atropine, scopolamine, apoatropine, 3α-tigloyloxytropane, 3,6-ditigloyloxytropane	B_2_; B_3_	[2,54,71,72]
*Scopolia*	*S. japonica* (L)	Japanese belladonna	Atropine, scopolamine	A_3_; A_5_; B_1_; B_2_; B_3_; B_4_; C_1_	[73]

* NR = no reports found. ^1^ Invasive weed potential in Europe: H = high, M = moderate, L = low, U = unknown.

**Table 3:** Foods involved in European Union Rapid Alert System for Food and Feed (RASFF) notifications for atropine and scopolamine; *Belladonna*, *Datura*, *Hyoscyamus*, *Mandragora* and *Solanum nigrum* in food (31 December 2022) [109].

Product Category/ Sub-Product Category as Used by RASFF	RASFF Reference Number	Contaminant (Tropane Alkaloid or Plant Part)
Cereals and bakery products		
Buckwheat	2021.4323; 2019.3045; 2018.3720; 2013.0829; 2013.0706; 2012.0794; 2009.0558; 2006.0424; 2006.BMT	Atropine, scopolamine
Corn-based food, popcorn	2022.4374; 2022.4371; 2022.3840; 2022.6084; 2021.0236; 2020.5394; 2019.1214; 2018.1447; 2016.0975; 2015.1190; 2015.0684; 2015.0210	Atropine, scopolamine
Millet and millet-based food	2021.1741; 2020.5696; 2020.3576; 2016.1298; 2015.0399; 2015.0388; 2015.0387; 2015.0339; 2015.0338; 2015.0203; 2014.1652 2006.0833; 2006.0737; 2006.CRE; 2006.COH; 2006.CFX	Atropine, scopolamine *Datura stramonium* seeds
Sorghum-based food	2016.0106; 2015.1487	Atropine, scopolamine
Flax seed meal	2021.6052	Atropine
Muesli	2018.2695	Atropine, scopolamine
Breakfast cereals	2020.2867	Atropine, scopolamine
Oatmeal	2020.5838	*Datura stramonium* seeds
Soy flakes	2020.0366	Atropine, scopolamine
Cocoa and cocoa preparations, coffee and tea		
Tea (herbal), blackberry leaves	2020.2159; 2017.0239; 2017.0153; 2016.1818; 1994.18 1984.03; 1983.03	Atropine, scopolamine *Atropa belladonna*
Peppermint	2019.0315	Atropine, scopolamine
Infusion	2021.6059	Atropine, scopolamine
Dietetic foods, food supplements, fortified foods		
Porridge (baby)	2016.0144; 2014.1596	Atropine
Millet and millet-based food	2014.1724; 2014.1694	Atropine
Dried herbs (*Ruscus aculeatus*)	2017.0803	NR *
Food additives and flavorings		
Soybean meal	2022.2074	Atropine, scopolamine
Fruits and vegetables		
Fresh spinach	*2022.5877*	*Mandragora*
Frozen spinach puree	2021.1390	Atropine, scopolamine
Vegetable mix	2019.0993; 2013.0696; 2007.CGO;	*Datura stramonium* seeds
Canned beans	2007.0613; 2006.0835;	*Datura stramonium* fruit
Frozen peas	2021.7140	*Solanum nigrum*
Canned tomatoes	2019.3340	*Datura stramonium* fruit
Herbs and spices		
Tea, herbal infusion, peppermint	2020.4733; 2018.2009 2013.0079; 1989.15	Atropine, scopolamine *Atropa belladonna*
Parsley stalks	2021.3836	Not mentioned
Savory	2022.2692	Atropine, scopolamine
Nuts, nut products and seeds		
Whole cumin seeds	2018.0774	Atropine, scopolamine
Poppy seeds	2008.0520; 2007.0267; 2007.0256	*Hyoscyamus niger* seeds
Feed, feed materials, pet food		
Sunflower or red millet seeds	2019.3256; 2019.0379; 2012.0354; 2006.BYZ	*Datura stramonium* seeds

* NR = no reports found.

**Table 4:** Reported cases of human intoxication in Europe.

Plant Involved	Product Involved	Reference
*Atropa belladonna*	Berries-accidental-Italy Berries-accidental-Denmark Berries-accidental-Netherlands Berries-accidental-Germany Berries, raw and cooked-accidental-France Tea, nettle-co-contamination-Austria Tea, comfrey-co-contamination-United Kingdom Tea, medicinal herb-Germany Tea, lungwort-co-contamination-Spain Tea, *Althea officinalis*-co-contamination-Netherlands	[111] [112] [113] [114] [115] [1,116] [117,118,119] [18] [119,120] [20,121]
*Datura* sp.	Buckwheat flour, bakery products-co-contamination-France	[19]
*Datura stramonium*	Buckwheat flour-co-contamination-Slovenia Buckwheat flour-co-contamination-France Millet flour/millet-carrot balls-co-contamination-Austria Green beans, canned-*Datura*-co-contamination-France Vegetables, mixed frozen-*Datura* seeds-co-contamination-Finland Frozen spinach puree-co-contamination-Slovakia (RASFF 2021.1390) Tea, *Datura* leaves-Germany *D. stramonium* leaves in pumpkin flower fritters-accidental-Italy	[17] [85] [23] [122] [21] [123] [124] [24]
*Datura innoxia*	Salad leaves, collected in the wild-co-contamination-Greece	[125]
*Mandragora officinarum* or *autumnalis*	Leaves-co-contamination-Greece Leaves-co-contamination-Italy Spinach-co-contamination-Italy	[22] [79] [80,81]

## Data Availability

No new data were created or analyzed in this study. Data sharing is not applicable to this article.

## Supplementary Materials

The following supporting information can be downloaded at: https://www.mdpi.com/article/10.3390/toxins15020098/s1, Figure S1. Biosynthetic pathways of tropanes from the amino acid ornithine. TR I = tropinone reductase I, TR II-tropinone reductase II, H6H: hyoscyamine 6β-hydroxylase, TAT = tropine acyltransferase, PAT = pseudotropine acyltransferase; Figure S2. Structures of atropine and scopolamine and related compounds; Figure S3. Structures of Convolvulaceae-type tropane esters; Figure S4. Structures of low molecular weight (LMW) tropane esters; Figure S5. Structures of LMW tropanes; Figure S6. Structures of calystegines; Table S1: Occurrence of calystegines in potato, eggplant and bell pepper plants from the Solanaceae family available at retail stores in Europe [31]; Table S2: Occurrence of TAs in single component flours, cereal-based food products and other products available at retail stores in Europe [31]; Table S3: RASFF notifications for tropane alkaloids atropine and scopolamine in food (31 December 2022) [109]; Table S4: RASFF notifications for Belladonna, Datura, Hyoscyamus and Solanum in food and feed (31 December 2022) [109].
