# Supplementary material for: Emerging Issues on Tropane Alkaloid Contamination of Food in Europe
Source: Toxins (Basel). 2023 Jan 19;15(2):98. doi: 10.3390/toxins15020098 (PMC9961018; doi:10.3390/toxins15020098)
Supplement: Supplementary file 1 [file toxins-15-00098-s001.zip › toxins-2104215-supplementary.pdf]

toxins

*Supplementary Materials*

# Emerging Issues on Tropane Alkaloid Contamination of Food in Europe

**Monique de Nijs, Colin Crews, Folke Dorgelo, Susan MacDonald and Patrick P. J. Mulder**

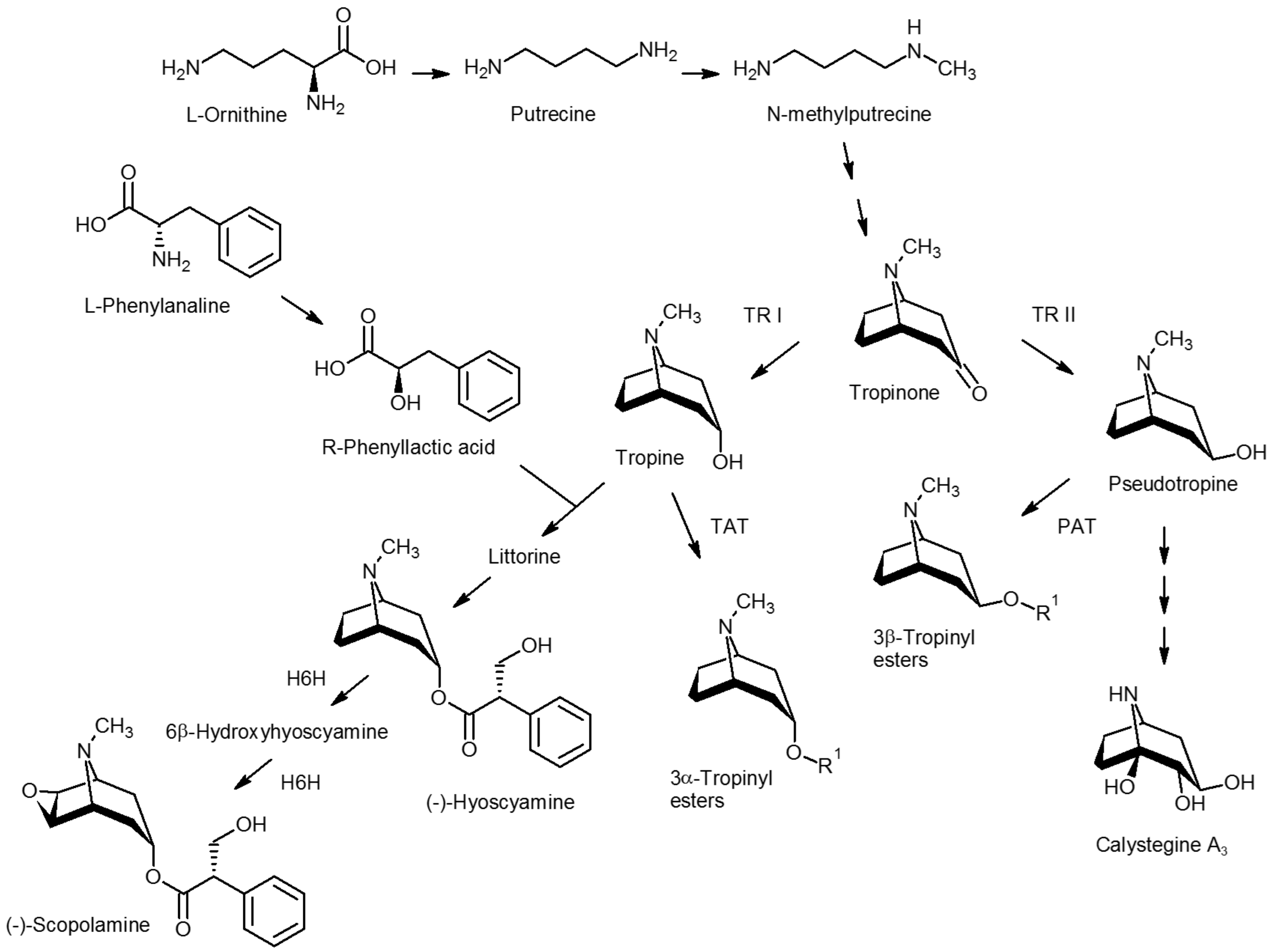


**Figure S1.** Biosynthetic pathways of tropanes from the amino acid ornithine.TR I = tropinone reductase I, TR II - tropinone reductase II, H6H: hyoscyamine 6β-hydroxylase, TAT = tropine acyltransferase, PAT = pseudotropine acyltransferase.

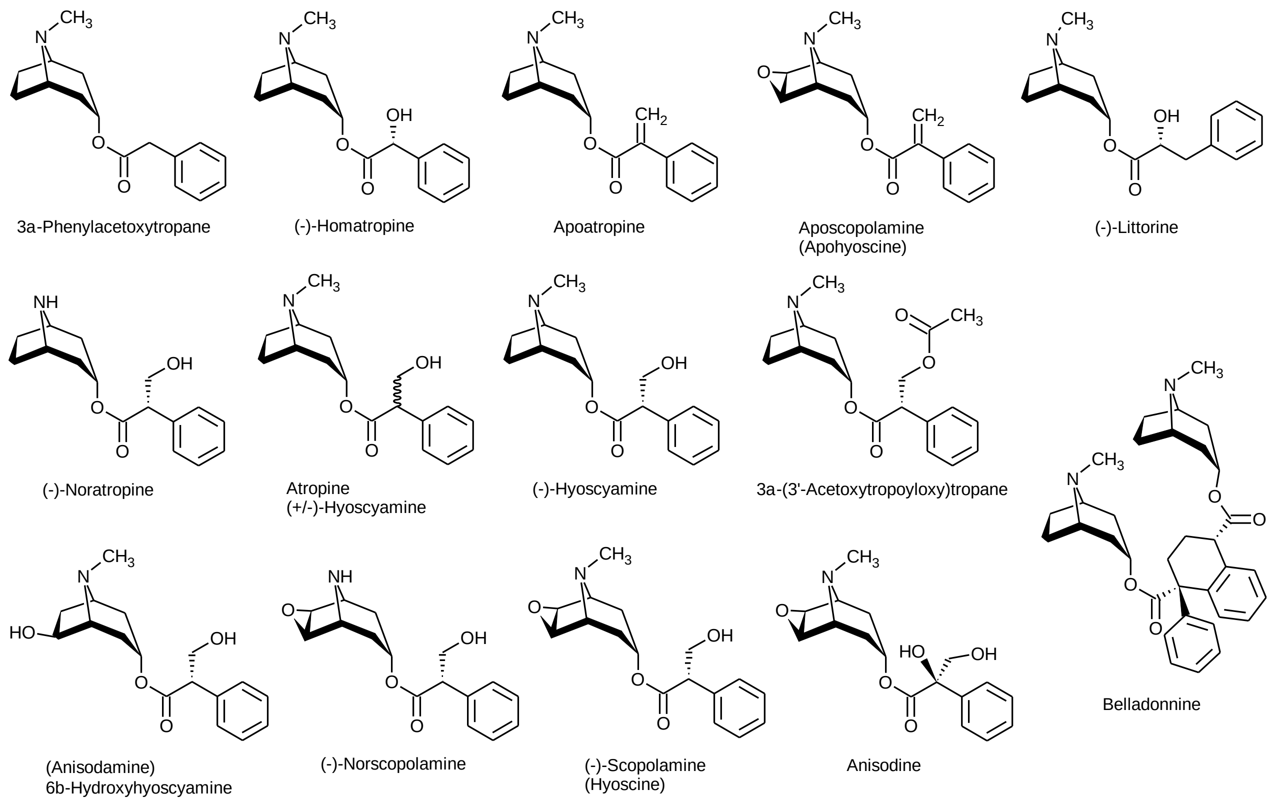


**Figure S2.** Structures of atropine and scopolamine and related compounds.

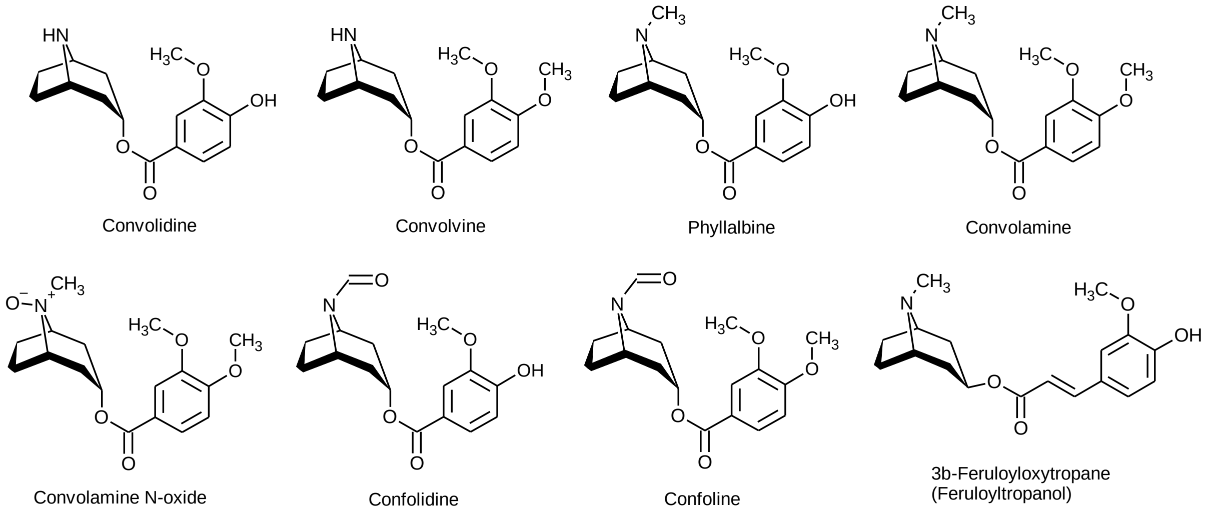


**Figure S3.** Structures of Convolvulaceae-type tropane esters

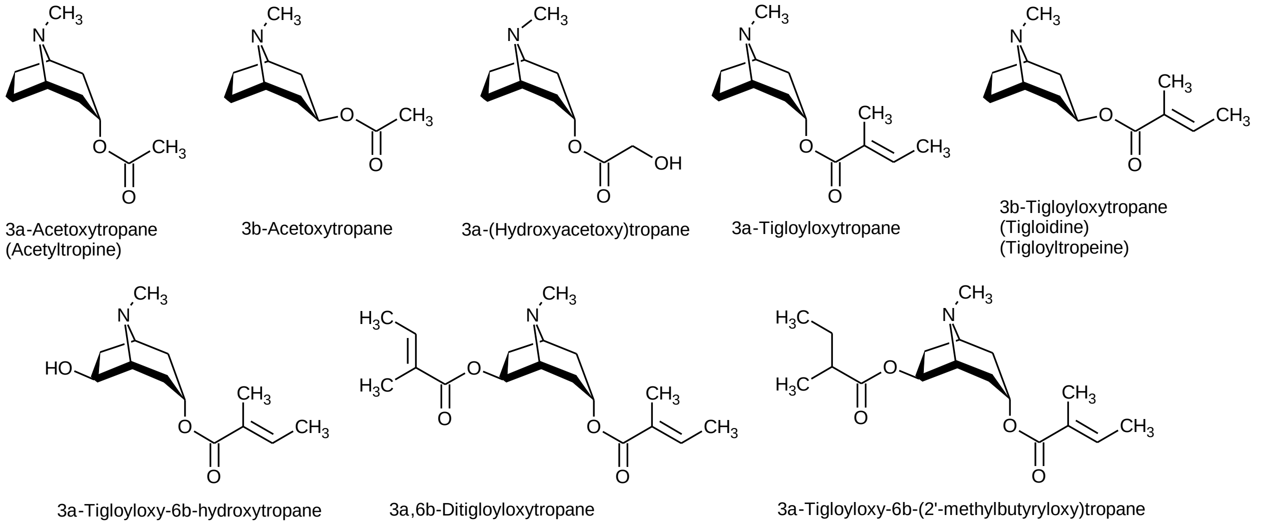


**Figure S4.** Structures of low molecular weight (LMW) tropane esters

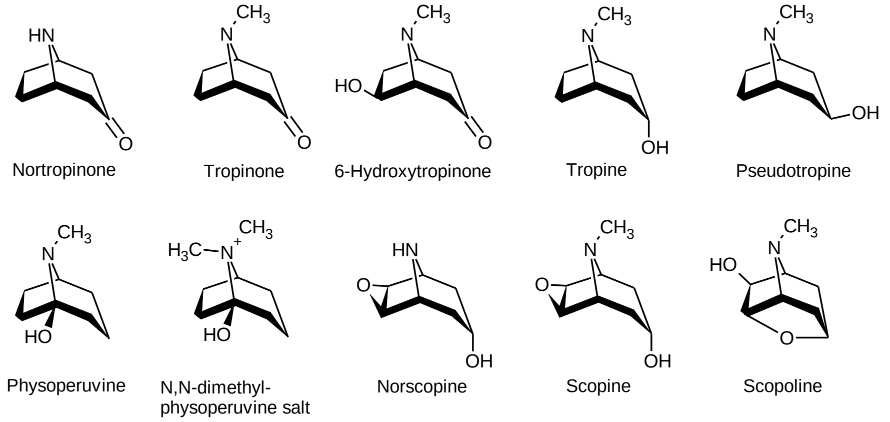


**Figure S5.** Structures of low molecular weight (LMW) tropanes.

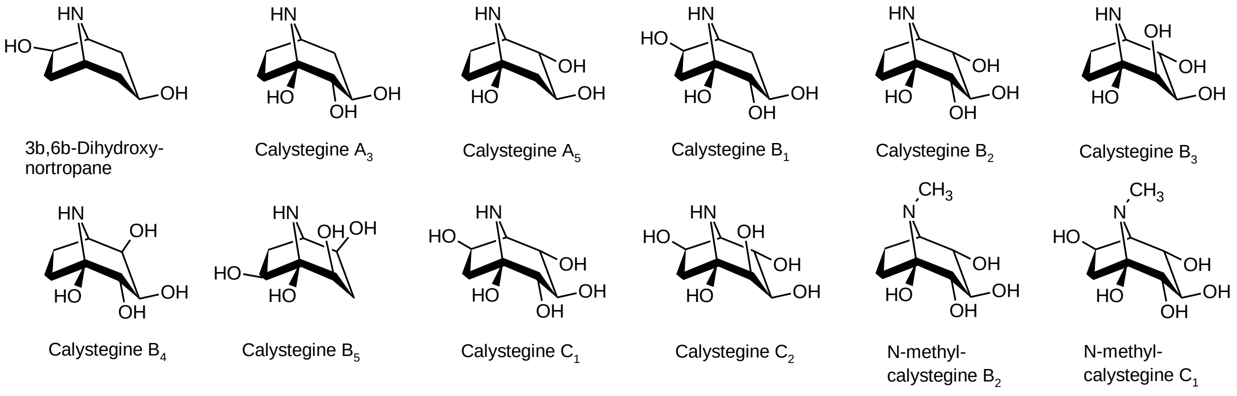


**Figure S6.** Structures of calystegines.

**Table S1.** Occurrence of calystegines in potato, eggplant and bell pepper plants from the Solanaceae family available at retail stores in Europe [31].

| Food category | Number of samples collected and analysed | % samples > LOD[1)] | Mean conc.[2)] (mg/kg f.w.[3)]) | Maximum conc. (mg/kg f.w.) | Calystegine, mean concentration (mg/kg f.w.) | | | | | |
|---|---|---|---|---|---|---|---|---|---|---|
| | | | | | $A_3$ | $A_5$ | $B_1$ | $B_2$ | $B_3$ | $B_4$ |
| *Potatoes* | 308 | 100.0% | 161.6 | 507.3 | | | | | | |
| Potatoes, fresh | 297 | 100.0% | 164.0 | 507.3 | 108.3 | 0.4 | n.d. | 52.1 | 0.1 | 3.7 |
| Potatoes, processed | 11 | 100.0% | 95.6 | 207.7 | 67.1 | n.d. | n.d. | 24.7 | 0.3 | 3.5 |
| *Eggplant & bell pepper* | 96 | 92.7% | 19.8 | 181.5 | | | | | | |
| Eggplant | 90 | 96.7% | 21.1 | 181.5 | 2.7 | n.d. | 3.9 | 14.5 | n.d. | n.d. |
| Bell pepper | 6 | 33.3% | 0.2 | 0.5 | n.d. | n.d. | 0.2 | n.d. | n.d. | n.d. |
| **Total Solanaceae food plants** | **404** | **98.3%** | **127.9** | **507.3** | | | | | | |

1) >LOD = higher than Limit of Detection
2) conc. = concentration
3) f.w. = fresh weight

**Table S2**. Occurrence of tropane alkaloids in single component flours, cereal-based food products and other products available at retail stores in Europe [31].

| Food category | Number of samples collected and analysed | All tropane alkaloids[1] | | | Atropine + scopolamine | | |
|---|---|---|---|---|---|---|---|
| | | % samples > LOD [2] | Mean conc.[3] (µg/kg) | Maximum conc. (µg/kg) | % samples > LOD[2] | Mean conc. (µg/kg) | Maximum conc. (µg/kg) |
| *Single component flours* | *268* | *21.3%* | *3.11* | *361.2* | *20.1%* | *2.87* | *334.8* |
| Buckwheat | 113 | 9.7% | 2.69 | 258.1 | 9.7% | 2.58 | 246.8 |
| Millet & sorghum | 102 | 23.5% | 5.15 | 361.2 | 22.5% | 4.67 | 334.8 |
| Corn & others | 53 | 20.8% | 0.07 | 2.2 | 18.9% | 0.03 | 0.8 |
| *Cereals-based products* | *838* | *14.0%* | *0.30* | *111.8* | *12.5%* | *0.25* | *108.5* |
| Bread and pasta | 195 | 9.2% | 0.06 | 4.2 | 7.7% | 0.04 | 4.2 |
| Bread | 114 | 15.8% | 0.10 | 4.2 | 13.2% | 0.07 | 4.2 |
| Pasta | 81 | 0.0% | 0.00 | 0.0 | 0.0% | 0.00 | 0.0 |
| Breakfast cereals | 219 | 6.8% | 0.63 | 111.8 | 5.9% | 0.59 | 108.5 |
| Biscuits and pastry | 164 | 14.6% | 0.14 | 12.0 | 13.4% | 0.06 | 2.3 |
| Biscuits | 150 | 14.7% | 0.14 | 12.0 | 13.3% | 0.05 | 2.3 |
| Pastry | 14 | 14.3% | 0.15 | 1.9 | 14.3% | 0.15 | 1.9 |
| Cereal-based foods for children | 260 | 20.0% | 9.49 | 859.5 | 14.2% | 0.09 | 4.2 |
| Breakfast cereals | 135 | 13.3% | 0.16 | 4.5 | 12.6% | 0.13 | 4.2 |
| Cookies | 107 | 13.1% | 0.85 | 86.2 | 11.2% | 0.03 | 0.8 |
| Pasta and cereal-based meals | 18 | 55.6% | 130.7 | 859.5 | 22.2% | 0.20 | 1.5 |
| *Other products available at retail stores* | *199* | *52.3%* | *73.35* | *4357.6* | *39.7%* | *8.15* | *428.5* |
| Dry (herbal) teas | 121 | 70.2% | 71.38 | 4357.6 | 63.6% | 13.40 | 428.5 |
| Legumes, stir-fry mixes, oil seeds | 78 | 24.4% | 76.42 | 2215.9 | 2.6% | 0.00 | 0.2 |
| Legumes, stir-fry mixes | 65 | 26.2% | 91.70 | 2215.9 | 0.0% | 0.00 | 0.0 |
| Oil seeds | 13 | 15.4% | 0.02 | 0.2 | 15.4% | 0.02 | 0.2 |
| **Total** | **1305** | **22.5%** | **12.90** | **4357.6** | **18.5%** | **1.96** | **428.5** |

1) Analysis of 24 tropane alkaloids, including atropine and scopolamine.

2) Products containing at least one tropane alkaloid above the Limit of Detection (LOD). LOD differs between components, matrices and laboratories.

3) conc. = concentration.

**Table S3.** RASFF notifications for tropane alkaloids atropine and scopolamine in food (December 31, 2022) [109]

| Type | Date of case | Reference | Notifying country | Product Category | Food | Country of origin | Concentration (μg/kg) | |
|---|---|---|---|---|---|---|---|---|
| | | | | | | | Atropine | Scopolamine |
| Alert notification[1)] | 27/7/2022 | 2022.4374 | Germany | Prepared dishes and snacks | Tortilla chips | Undecided | NR* | |
| Alert notification | 27/7/2022 | 2022.4371 | Germany | Prepared dishes and snacks | Tortilla chips | Belgium | NR | |
| Alert notification | 1/7/2022 | 2022.3840 | Germany | Prepared dishes and snacks | Corn chips | Belgium | NR | |
| Alert notification | 6/5/2022 | 2022.2692 | Germany | Herbs and spices | Savory | Austria | Sum 106$\pm$21.2 | |
| Alert notification | 7/4/2022 | 2022.2074 | Germany | Food additives and flavorings | Soybean meal | Austria | 56 | - |
| Alert notification | 9/11/2021 | 2021.6084 | Belgium | Cereals and bakery products | Corn flour | Belgium | 25.12 | 6.50 |
| Information notification for follow-up | 9/11/2021 | 2021.6059 | Belgium | Cocoa and cocoa preparations, coffee and tea | Infusion | Netherlands | 184 | 21 |
| Alert notification | 8/11/2021 | 2021.6052 | Germany | Cereals and bakery products | Organic flaxseed meal | Hungary | 100-238 | - |
| Notification | 12/08/2021 | 2021.4323 | Germany | Other food product/ mixed | Buckwheat flour, extruded | Austria/Ger many | 240+97 | 87+35 |
| Alert | 20/07/2021 | 2021.3836 | Germany | Herbs and spices | Parsley stalks, broken | Hungary | NR | NR |
| Alert | 07/04/2021 | 2021.1741 | Germany | Cereals and bakery products | Millet | the Netherlands | 40.1 | 29.1 |
| Alert | 17/03/2021 | 2021.1390 | Slovakia | Fruits and vegetables | Frozen spinach puree | Slovakia | 850-3,446 | 1,033-3,860 |
| Alert | 18/01/2021 | 2021.0236 | Germany | Cereals and bakery products | Popcorn maize | Spain | 23, 35 | 1.2, 1.9 |
| Information | 11/12/2020 | 2020.5696 | Germany | Cereals and bakery products | Wild ground brown millet | Germany | 36 | 22.4 |
| Alert | 27/11/2020 | 2020.5394 | Belgium | Cereals and bakery products | Maize for popcorn | Belgium | - | 14.6 |
| Information | 04/11/2020 | 2020.4733 | Germany | Herbs and spices | Peppermint | Turkey | 26.8 | 39.9 |
| Information | 04/09/2020 | 2020.3576 | Germany | Cereals and bakery products | Wild brown millet | Germany | 24.2 | 13.3 |
| Information | 25/05/2020 | 2020.2159 | Germany | Cocoa and cocoa preparations, coffee and tea | Blackberry leaves | Bulgaria | 543.1 | 31.4 |
| Alert | 24/01/2020 | 2020.0366 | Germany | Cereals and bakery products | Soy flakes | Czech Republic | 19 | 6.4 |
| Alert | 26/08/2019 | 2019.3045 | France | Cereals and bakery products | Buckwheat | France | 47 | 30 |
| Alert | 06/08/2019 | 2019.2867 | Netherlands | Cereals and bakery products | Breakfast porridge 8 cereals with honey | Spain | 2.2 | 2.7 |

| Type | Date of case | Reference | Notifying country | Product Category | Food | Country of origin | Concentration (µg/kg) | |
|---|---|---|---|---|---|---|---|---|
| | | | | | | | Atropine | Scopolamine |
| Information | 01/04/2019 | 2019.1214 | Croatia | Cereals and bakery products | Corn grits | Serbia | 4.5 | 4.3 |
| Information | 29/01/2019 | 2019.0315 | Croatia | Cocoa and cocoa preparations, coffee and tea | Peppermint | Serbia | 200.5 | 488.7 |
| Alert | 20/12/2018 | 2018.3720 | France | Cereals and bakery products | Organic buckwheat flour | France | 53 | 20 |
| Alert | 26/09/2018 | 2018.2695 | Germany | Cereals and bakery products | Organic muesli | Austria | 60.7 | 38.8 |
| Alert | 16/07/2018 | 2018.2009 | Czech Republic | Herbs and spices | Herbal infusion | Poland | 213 | 44.7 |
| Alert | 24/05/2018 | 2018.1447 | Germany | Cereals and bakery products | Popcorn | France | 6.60 | 1.77 |
| Information | 21/03/2018 | 2018.0774 | Hungary | Nuts, nut products and seeds | Whole cumin seeds | Hungary | 16,177.6 | 4,658.3 |
| Alert | 07/06/2017 | 2017.0803 | United Kingdom | Dietetic foods, food supplements, fortified foods | Dried herbs (*Ruscus aculeatus*) | Bulgaria | NR | NR |
| Alert | 24/02/2017 | 2017.0239 | Czech Republic | Cocoa and cocoa preparations, coffee and tea | Herbal tea with Echinacea | Poland | 72 | 23 |
| Alert | 06/02/2017 | 2017.0153 | Germany | Cocoa and cocoa preparations, coffee and tea | Organic burdock root (*Arctium lappa*) tea | Albania/ Croatia | 520 | NR |
| Alert | 28/12/2016 | 2016.1818 | Czech Republic | Cocoa and cocoa preparations, coffee and tea | Herbal tea | Poland | 206.4 | 31.7 |
| Alert | 20/09/2016 | 2016.1298 | Austria | Cereals and bakery products | Millet flour | Hungary | 23.5 | 9.5 |
| Alert | 22/07/2016 | 2016.0975 | Austria | Cereals and bakery products | Corn | Germany | 10.5; 12.2; 12.3 | 2.0; 3.8; 3.1 |
| Alert | 13/04/2016 | 2016.0144 | Czech Republic | Dietetic foods, food supplements, fortified foods | Baby porridge without milk | Spain | 7.9 | NR |
| Alert | 01/02/2016 | 2016.0106 | Czech Republic | Cereals and bakery products | Gluten-free baking mix based on sorghum | Czech Republic | 180; 130 | 36; 27 |
| Alert | 26/11/2015 | 2015.1487 | Czech Republic | Cereals and bakery products | Sorghum flour | Czech Republic/ Slovakia | 1,200; 1,500 | 360; 460 |
| Alert | 18/09/2015 | 2015.1190 | Germany | Cereals and bakery products | Microwave popcorn | Spain | 29 | 6 |
| Alert | 04/06/2015 | 2015.0684 | Germany | Cereals and bakery products | Organic polenta cornmeal | Germany | 198.5 | 45 |
| Alert | 31/03/2015 | 2015.0399 | Germany | Cereals and bakery products | Millet balls | Hungary | 384 | 388 |

| Type | Date of case | Reference | Notifying country | Product Category | Food | Country of origin | Concentration (µg/kg) | |
|---|---|---|---|---|---|---|---|---|
| | | | | | | | Atropine | Scopolamine |
| Alert | 30/03/2015 | 2015.0388 | Austria | Cereals and bakery products | Gluten free organic millet | Austria | 30 | 24 |
| Alert | 30/03/2015 | 2015.0387 | Austria | Cereals and bakery products | Millet honey poppies | Germany | 26 | 11 |
| Alert | 20/03/2015 | 2015.0339 | Austria | Cereals and bakery products | Millet dumplings | Hungary | 304 | 358 |
| Alert | 20/03/2015 | 2015.0338 | Austria | Cereals and bakery products | Organic millet dumplings | Hungary | 481 | 533 |
| Alert | 20/02/2015 | 2015.0210 | Germany | Cereals and bakery products | Organic polenta cornmeal | Germany | 156.2; 207.5 | 27.2; 31.3 |
| Alert | 20/02/2015 | 2015.0203 | Austria | Cereals and bakery products | Brown millet | Germany | 62 | 33 |
| Alert | 18/12/2014 | 2014.1724 | Germany | Dietetic foods, food supplements, fortified foods | Millet/cereal porridge with rice | Hungary | 36.6 | NR |
| Alert | 11/12/2014 | 2014.1694 | Germany | Dietetic foods, food supplements, fortified foods | Organic baby food apple pear millet | Germany | 3.7; 6.7; 31.9 | NR |
| Alert | 04/12/2014 | 2014.1652 | Germany | Cereals and bakery products | Brown millet flour | Austria/ Hungary/ the Netherlands | 46 | 25 |
| Alert | 21/11/2014 | 2014.1596 | Germany | Dietetic foods, food supplements, fortified foods | Baby food porridge | Germany/ Austria | 12.1 | NR |
| Alert | 13/06/2013 | 2013.0829 | Slovenia | Cereals and bakery products | Buckwheat flour | Austria/ Slovakia | 18 | 5.5 |
| Alert | 22/05/2013 | 2013.0706 | Slovenia | Cereals and bakery products | Buckwheat flour | Czech Republic | 14 | 11 |
| Information | 08/06/2012 | 2012.0794 | Slovenia | Cereals and bakery products | Buckwheat flour | Slovenia | 20 | 6.1 |
| Information | 30/04/2009 | 2009.0558 | Slovenia | Cereals and bakery products | Buckwheat flour | Hungary | 110 | 47 |
| Information | 03/07/2006 | 2006.BMT | Slovenia | Cereals and bakery products | Buckwheat flour | Ukraine | 37 | 48 |
| Alert | 03/07/2006 | 2006.0424 | Slovenia | Cereals and bakery products | Buckwheat flour | Czech Republic | 35 | 65 |
| Alert | 28/10/1994 | 1994.18 | United Kingdom | Cocoa, coffee and tea | Burdock root tea | United Kingdom | NR | NR |

* NR = not reported.

1) updated version of the RASSF portal from mid-2021 makes no difference between alert or notification and Alert notification.

**Table S4.** RASFF notifications for Atropa, Belladonna, Datura, Hyoscyamus, Mandragora and Solanum in food and feed (December 31, 2022) [109]

| Type | Date of case | Reference | Notifying country | Product Category | Food | Country of origin | Contaminant plant | Concentration |
|---|---|---|---|---|---|---|---|---|
| Alert[1)] | 23/01/2013 | 2013.0079 | The Netherlands | Herbs and spices | Marshmallow tea | Germany/ Bulgaria | *A. belladonna* root | NR* |
| Alert | 29/06/1989 | 1989.15 | France | Herbs and spices | Burdock root | NM | Belladonna | NR |
| Alert | 23/01/1984 | 1984.03 | France | Cocoa, coffee and tea | Bardane herbal tea | NM | Belladonna | NR |
| Alert | 04/02/1983 | 1983.03 | United Kingdom | Cocoa, coffee and tea | Tea | NM | Belladonna | NR |
| Alert | 17/12/2020 | 2020.5838 | Hungary | Cereals and bakery products | Grain oatmeal | Poland | *D. stramonium* seeds | NR |
| Alert | 20/09/2019 | 2019.3340 | France | Fruits and vegetables | Canned peeled tomatoes | Spain | *D. stramonium* fruits | NR |
| Alert | 12/09/2020 | 2019.3256 | Austria | Compound feeds | Seedmix for birds | The Netherlands | *D. stramonium* seeds | 0.1235% |
| Alert | 15/03/2019 | 2019.0993 | France | Fruits and vegetables | Frozen beans | France | *D. stramonium* seeds | NR |
| Information | 01/02/2019 | 2019.0379 | Belgium | Feed material | Sunflower seeds | The Netherlands | *D. stramonium* seeds | 0.12% |
| Alert | 17/05/2013 | 2013.0696 | Finland | Fruits and vegetables | Frozen vegetable-bean-seed mix | Belgium/ Spain | *D. stramonium* seeds | NR |
| Information | 07/03/2012 | 2012.0354 | Denmark | Bird feed | Sunflower seeds | France | *D. stramonium* seeds | 0.1862% |
| Information | 02/10/2007 | 2007.CGO | Spain | Fruits and vegetables | Vegetable bacon stir-fry | Spain | *D. stramonium* seeds | NR |
| Alert | 05/09/2007 | 2007.0613 | Austria | Fruits and vegetables | Canned green beans | Hungary | *D. stramonium* fruit | NR |
| Alert | 27/11/2006 | 2006.0835 | Austria | Fruits and vegetables | Canned green beans | Hungary | *D. stramonium* fruit | NR |
| Alert | 27/11/2006 | 2006.0833 | Austria | Cereals and bakery products | Organic millet | Austria/ Hungary | *D. stramonium* seeds | 130 seeds/kg |
| Information | 20/11/2006 | 2006.CRE | Austria | Cereals and bakery products | Brown millet | Austria | *D. stramonium* seeds | NR |
| Information | 08/11/2006 | 2006.COH | Austria | Cereals and bakery products | Organic millet | Austria | *D. stramonium* seeds | NR |
| Alert | 24/10/2006 | 2006.0737 | Austria | Cereals and bakery products | Organic millet | Austria | *D. stramonium* seeds | NR |
| Information | 10/10/2006 | 2006.CFX | Austria | Cereals and bakery products | Peeled bio gold millet | Austria | *D. stramonium* seeds | NR |
| Information | 28/08/2006 | 2006.BYZ | Germany | Pet food | Red millet seeds | Hungary | *D. stramonium* seeds | 0.276% |
| Alert | 06/05/2008 | 2008.0520 | Czech Republic | Nuts, nut products and seeds | Poppy seeds | Czech Republic | *H. niger* seeds | 0.13% |
| Alert | 13/04/2007 | 2007.0267 | Slovakia | Nuts, nut products and seeds | Poppy seeds | Czech Republic | *H. niger* seeds | 0.24% |
| Alert | 10/04/2007 | 2007.0256 | Slovakia | Nuts, nut products and seeds | Poppy seeds | Czech Republic | *H. niger* seeds | 0.42% |

| Type | Date of case | Reference | Notifying country | Product Category | Food | Country of origin | Contaminant plant | Concentration |
|---|---|---|---|---|---|---|---|---|
| Alert notification | 10/10/2022 | 2022.5877 | Italy | Fruits and vegetables | Fresh spinach | Italy | *Mandragora* | NR |
| Alert notification | 24/12/2021 | 2021.7140 | Belgium | Fruits and vegetables | Frozen peas | Belgium | *S. nigrum* | NR |

* NR = not reported.

1) updated version of the RASSF portal from mid-2021 makes no difference between alert or notification and alert notification.
